# The role of ultra-processed foods in plant-based diets: associations with human health and environmental sustainability

**DOI:** 10.1007/s00394-024-03477-w

**Published:** 2024-08-24

**Authors:** Merel C. Daas, Reina E. Vellinga, Maria Gabriela M. Pinho, Jolanda M. A. Boer, W. M. Monique Verschuren, Yvonne T. van der Schouw, Pieter van’t Veer, Sander Biesbroek

**Affiliations:** 1 Division of Human Nutrition and Health, Wageningen University & Research, P.O. Box 17, Wageningen, 6700 AA The Netherlands; 2 Centre for Prevention, Lifestyle and Health, National Institute for Public Health and the Environment (RIVM), Antonie van Leeuwenhoeklaan 9, Bilthoven, 3721 MA The Netherlands; 3 Copernicus Institute of Sustainable Development, Utrecht University, Princetonlaan 8a, Utrecht, 3584 CB The Netherlands; 4 Julius Center for Health Sciences and Primary Care, University Medical Center Utrecht, Universiteitsweg 100, Utrecht, 3584 CG The Netherlands

**Keywords:** Plant-based diet, Ultra-processed foods, NOVA classification, All-cause mortality, Environmental impact, Cohort study

## Abstract

**Purpose:**

Investigate the associations of ultra-processed foods (UPF) in healthful (hPDI) and unhealthful (uPDI) plant-based diets with all-cause mortality, greenhouse gas emissions (GHGE), and blue water consumption (BWC).

**Methods:**

Analyses were based on 35,030 participants (20–70 years; 74% females) from the EPIC-NL cohort who were followed up from 1993 to 1997 through 2014. Plant-based diet indices (hPDI and uPDI) and UPF consumption were calculated from a validated FFQ, assessed at baseline. Cox proportional hazard and multiple linear regression models were used to estimate associations between combined quartiles of the PDI indices and UPF consumption.

**Results:**

With lower hPDI and higher UPF diets as the reference, we observed the following. Risk estimates of all-cause mortality were 0.98 (95% CI: 0.83, 1.16) for lower UPF consumption, 0.86 (95% CI: 0.68, 1.08) for higher hPDI, and 0.78 (95% CI: 0.66, 0.89) for combined higher hPDI and lower UPF consumption. Results with the uPDI were inconclusive. Mean differences in GHGE and BWC were 1.4% (95% CI: 0.3, 2.4) and 1.6% (95% CI: -0.5, 3.7) for lower UPF consumption, -7.4% (95% CI: -8.6, -6.4) and 9.6% (95% CI: 7.2, 12.0) for higher hPDI, and − 6.8% (95% CI: -7.4, -6.1) and 13.1% (95% CI: 11.6, 14.8) for combined higher hPDI and lower UPF consumption. No apparent conflict between environmental impacts was observed for the uPDI; GHGE and BWC were lower for higher uPDI scores.

**Conclusion:**

Mortality risk and environmental impacts were mostly associated with the amount of plant-based foods and to a lesser extent UPF in the diet. Shifting to a more healthful plant-based diet could improve human health and reduce most aspects of environmental impact (GHGE, but not BWC) irrespective of UPF consumption.

**Supplementary Information:**

The online version contains supplementary material available at 10.1007/s00394-024-03477-w.

## Introduction

There is growing evidence for the impact of our food choices on health and the environment [1, 2]. Western diets, high in animal-based foods, are recognized as a leading risk factor of non-communicable diseases and premature mortality [2–5]. Many studies have demonstrated associations between a high consumption of red and processed meat and increased incidences of cardiovascular disease, type 2 diabetes, and certain types of cancer, as well as mortality rates [6–10]. Besides, current food production and consumption patterns are estimated to contribute to 20–30% of total greenhouse gas emissions (GHGE) and are a major cause of biodiversity loss, deforestation, and water extraction and pollution [11–14]. Particularly diets high in animal-based foods play a substantial role herein [15, 16]. Meat and dairy production accounts for the largest share of environmental impact due to the inefficient conversion of animal feed into food, causing 50% of GHGE in the food sector and using 80% of total farmlands [17].

A transition towards a plant-based diet, focusing on a high consumption of plant foods and low to no consumption of animal-based foods, has been of interest to provide benefits for both health and the environment [18–21]. Several modelling studies that replaced animal-based foods by foods of plant origin have shown large reductions in GHGE and land use, and a lower risk of all-cause mortality [22–25]. Despite these positive effects, it should be considered that various plant foods differ in terms of environmental impact and nutritional quality. For instance, several plant foods, such as nuts, fruits, and vegetables, have been associated with a relatively high consumption of blue water as these foods require high amounts of irrigation water to grow [26–29]. Moreover, earlier studies revealed that the associations between plant-based diets and health depend on the types of plant foods consumed [18, 30–36]. Greater adherence to a diet high in healthy plant foods (e.g. whole grains, vegetables, legumes, etc.) was shown to be associated with reduced incidences of cardiovascular disease and mortality rates, while individuals who consumed mostly unhealthy plant foods (e.g. sweetened beverages, fries, sweets, etc.) were at increased risk [30, 33, 35, 36].

As many of these unhealthy plant foods can be classified as ultra-processed foods (UPF), the adverse effects of unhealthy plant-based diets may be due to the high amount of UPF in these diets. UPF are defined as formulations of ingredients derived from foods and additives, that result from various processing steps during which substances are added to enhance shelf-life and palatability [37]. These foods often have a higher content of added sugars, saturated fats, and sodium, and contain reduced amounts of protein, fiber, and micronutrients [38, 39]. Considering that most of these nutritional factors are directly related to cardiometabolic health, a higher consumption of UPF has been associated with an increased risk of non-communicable diseases and mortality rates [40–42]. In addition to the adverse health outcomes, UPF are thought to account for more than a third of environmental effects caused by the food sector due to the high number of processing steps, over-packaging, and longer transport distances [43, 44]. During the past decades, the global consumption of UPF has increased substantially, with vegetarians and vegans consuming even higher amounts than meat-eaters [5, 45–48]. A recent study reported that specifically individuals who consume unhealthy plant-based diets have a higher consumption of UPF compared to those who consume mostly healthy plant-based diets [49]. Given a potential substitution of animal-based foods with UPF, these findings suggest that the health and environmental benefits of a plant-based diet may depend on the degree of processing of the foods consumed.

While earlier research has established a solid foundation for the beneficial associations of plant-based diets with human and planetary health [2, 15, 18], associations with plant-based UPF have been less explored. Therefore, this study aimed to investigate the associations of UPF in healthful and unhealthful plant-based diets with all-cause mortality, GHGE, and blue water consumption (BWC).

## Methods

### Study design and population

Data from the Dutch contribution to the European Prospective Investigation into Cancer and Nutrition (EPIC) study was used for the analysis. The EPIC study is a population-based, prospective cohort that was initiated in ten European countries to study the role of diet and physical activity in the etiology of cancer and other chronic diseases [50]. The Dutch contribution (EPIC-NL) includes the Prospect cohort and MORGEN cohort and in total consisted of 40,011 participants recruited between 1993 and 1997 [51]. The Prospect cohort comprised 17,357 females aged 49–70 years who participated in a regional breast cancer screening program in the city of Utrecht and its surroundings [52]. The MORGEN cohort included 22,654 males and females aged 20–65 years who were randomly selected from the general population of Amsterdam, Maastricht, and Doetinchem [53, 54]. The EPIC-NL study complies with the guidelines described in the Declaration of Helsinki, and all procedures involving human participants were approved by the Institutional Review Board of the University Medical Centre Utrecht and the Medical Ethical Committee of TNO Nutrition and Food Research. All participants provided their written informed consent [51].

For the present analyses, exclusion criteria were missing dietary information at baseline (*n* = 217), no informed consent for follow-up of vital status (*n* = 925), withdrawal of informed consent during follow-up (*n* = 1), and missing follow-up data of vital status (*n* = 142). Participants with a history of cancer (*n* = 1627), diabetes (*n* = 718), myocardial infarction (*n* = 427), or stroke (*n* = 361) at baseline were excluded because their usual reported diet may be influenced by their condition and not reflect their diet before diagnosis. To exclude implausible dietary values that could lead to incorrect analysis of the data, participants in the highest and lowest 0.5% of the reported energy intake to basal metabolic rate ratio (*n* = 332) were left out as well. Finally, participants with missing information on possible confounders, including BMI (*n* = 17), educational level (*n* = 191), or smoking status (*n* = 23), were excluded. After these exclusions, in total 35,030 participants remained for the complete-case analysis.

### Dietary assessment

Food consumption was measured at baseline (1993–1997) using a self-administered semi-quantitative food frequency questionnaire (FFQ), including questions on the usual frequency of consumption of 77 main food categories during the year before enrolment. The questionnaire allowed estimation of the usual daily dietary intake of 178 food items, and had been validated against twelve 24-h recalls and biomarkers in 24-h urine and serum [55, 56]. Spearman rank correlation coefficients based on estimates of the FFQ and 24-h recalls were 0.51 for potatoes, 0.36 for vegetables, 0.68 for fruits, 0.39 for meat, 0.69 for dairy, 0.76 for sugar and sweet products, and 0.52 for biscuits and pastry in males. Similar results were obtained for females. Nutrient intakes were calculated using the 1996 Dutch Food Composition Table [57].

### Plant-based diet indices

In order to assess participants’ adherence to a healthy and less healthy plant-based diet, the healthful plant-based diet index (hPDI) and unhealthful plant-based diet index (uPDI) were calculated based on the procedure used by Martínez-González et al. [58] and adapted by Satija et al. [32]. Supplemental Table 1 displays the food groups with included foods and scoring criteria for the two PDI indices. First, 18 food groups were created based on nutrient and culinary similarities within the larger categories of healthy plant-based foods (vegetables, fruit, legumes, whole grains, nuts and seeds, vegetable oils and fats, and tea and coffee), unhealthy plant-based foods (refined grains, potatoes, juices, (sugar) sweetened beverages, and sweets and desserts), and animal-based foods (meat, animal fats, eggs, fish and seafood, dairy products, and miscellaneous animal-based foods). All FFQ items were assigned to the appropriate food group and checked by a research dietician. We distinguished between healthy and unhealthy plant-based foods using the most recent empirical evidence on their associations with several chronic conditions (i.e. obesity, hypertension, lipids, inflammation, type 2 diabetes, cardiovascular disease, and certain cancers) [32]. Alcoholic beverages were not included in the indices due to differential associations with health outcomes, but included as a covariate in the analyses. Mixed dishes that contain substantial amounts of animal sourced ingredients (e.g. soups, pizza, and mayonnaise) were classified as miscellaneous animal-based foods, in accordance with previous studies [30–34].

Second, food consumption in g/day of each food group was calculated for all individuals by summing the consumption of the FFQ items. Within each food group, participants were classified into quintiles according to their consumption of that specific food group after adjusting for total energy intake using the residual method. Based on the quintiles within each food group, participants were given a positive score between 1 (lowest quintile) and 5 (highest quintile) or reverse score between 5 (lowest quintile) and 1 (highest quintile). For the hPDI, healthy plant-based food groups were assigned positive scores and unhealthy plant-based and animal-based food groups were assigned reverse scores. For the uPDI, unhealthy plant-based food groups were given positive scores and healthy plant-based and animal-based food groups were given reverse scores. To obtain the PDI indices, the 18 food group scores for each individual were summed and could range from 18 (lowest possible score) to 90 (highest possible score).

Overall, the hPDI and uPDI give more points to high consumers of healthy and unhealthy plant-based food groups, respectively. It should be noted that high PDI indices do not equal a vegetarian or vegan diet, but rather indicate a relatively high consumption of plant-based foods and/or relatively low consumption of animal-based foods compared to the total study population. The indices are thus dependent on the food consumption in our study population and cannot be directly compared to other populations where the overall consumption of plant-based or animal-based foods may be higher or lower.

### UPF consumption

The NOVA classification was applied to assess the degree of food processing of the diet [37]. This classification includes four classes: unprocessed/minimally processed foods (MPF), processed culinary ingredients (PCI), processed foods (PF), and ultra-processed foods (UPF). To discriminate different foods between these classes, the NOVA classification takes into consideration the ingredient list of food items and all physical, chemical, and biological methods used during the food production process. An extensive description of the different classes can be found elsewhere [37]. All food items of the FFQ were previously assigned to one of the four classes of the NOVA classification based on the degree of processing [59]. To account for potential changes in food processing over time, three scenarios (lower, middle, and upper bound) were considered when classifying food items. The lower bound scenario encompassed food items that could have been less processed compared to the middle bound scenario and were assigned to a less processed NOVA class, whereas food items that could have been more processed were included in the upper bound scenario and assigned to a more processed NOVA class. The middle bound scenario mostly resembled with the dietary assessment that was conducted in the nineties and was therefore used in all analyses.

The daily consumption in g/2000 kcal of MPF, PCI, PF, and UPF was calculated for each participant by summing the consumption of the FFQ items for each NOVA class and dividing these by the total energy consumed per individual multiplied by 2000. A weight ratio instead of an energy ratio was used to account for food that does not provide energy (e.g. artificially sweetened beverages) and non-nutritional substances related to food processing (e.g. additives). In the primary analysis, alcoholic beverages were included in the NOVA classes to adhere as much as possible to the original classification. Sensitivity analyses in which alcoholic beverages were excluded from the calculation of the NOVA classes did not generate considerably different results (Supplemental Table 2). For the purposes of this study, we focused on UPF consumption from the NOVA classification as this was our main interest.

### Covariate assessment

At baseline, several lifestyle factors were assessed using a general questionnaire, containing questions on demographic characteristics, presence of chronic diseases, and related potential risk factors. Weight and height were measured by trained staff according to standardized protocols [51] and BMI was calculated by dividing weight by height squared (kg/m^2^). Duration and types of physical activity were assessed with a validated questionnaire [60] and classified according to the Cambridge Physical Activity Index (CPAI) with imputed data for missing values [61]. The CPAI was divided into four different categories: inactive, moderately inactive, moderately active, and active. Smoking was classified as never, former, and current smoker. Educational level was coded as low (lower vocational training or primary school), medium (intermediate vocational training or secondary school), or high (higher vocational training or university).

### Environmental impact

Environmental impact of the foods consumed was derived from the Dutch Life Cycle Assessment (LCA) food database [62] and has previously been described in more detail [29]. In short, the LCA methodology was applied to quantify the environmental impact for six different indicators (land use, BWC, GHGE, acidification, freshwater eutrophication, and marine eutrophication) throughout the entire foods’ life cycle. All life cycle stages from cradle to grave were included in the analyses, including primary production, processing, primary packaging, distribution, supermarket, retail, storage, preparation by the consumer, and waste or losses. Transport was only included from primary production to supermarket and food waste was calculated by using food group-specific percentages for avoidable and unavoidable food losses throughout the food chain. When production processes led to more than one food product, environmental impact was divided using economic allocation, except for milk where biophysical allocation was applied.

The LCA data were available for 242 foods that were selected based on frequency and quantity of consumption in the Dutch National Food Consumption Survey or its relatively high environmental impact per kg of food. These data were in prior linked to the EPIC-NL FFQ data [63]. For FFQ items of which primary data was not available, extrapolations were carried out by the Dutch National Institute for Public Health and the Environment (RIVM) based on similarities in type of food, production method, and ingredient composition. The LCAs are based on current production practices and are assumed to be equal in the nineties when food consumption was assessed. Since a previous study measured high correlations between GHGE and several environmental indicators (land use, acidification, fresh water eutrophication, and marine eutrophication), with exception of BWC, this study focused on GHGE and BWC to assess environmental impact [29]. BWC refers to the total volume of water sourced from surface and groundwater, as defined by Hoekstra et al. [64]. For each participant, the daily GHGE (kg CO_2_-eq) and daily BWC (L) were calculated in absolute and standardized amounts. Standardized amounts were divided by total energy intake and expressed per 2000 kcal.

### Mortality assessment

Vital status of all participants was obtained through linkage with the municipal population registries of the Netherlands. Participants were followed up over time until death from any cause, migration, loss to follow-up, or were censored. All-cause mortality was defined as death from any cause after study inclusion. Follow-up was completed on December 31st, 2014.

### Statistical analysis

Interaction between the PDI indices and UPF consumption in the associations with all-cause mortality and environmental impact were evaluated following the recommendations of Knol and VanderWeele [65]. First, participants were divided into sixteen dietary categories based on quartiles of the hPDI and quartiles of UPF consumption. The most extreme quartiles were mainly of interest: (1) low hPDI score, high UPF consumption (Q1hPDI/Q4UPF), (2) low hPDI score, low UPF consumption (Q1hPDI/Q1UPF), (3) high hPDI score, high UPF consumption (Q4hPDI/Q4UPF), and (4) high hPDI score, low UPF consumption (Q4hPDI/Q1UPF). For example, the Q1hPDI/Q4UPF category represented diets that were low in healthy plant-based foods (hPDI ≤ 50 points) and high in UPF (UPF > 433 g/2000 kcal), and was taken as a reference for all analyses. The same procedure was applied to the scores of the uPDI.

Cox proportional hazard models were used to estimate hazard ratios (HRs) with 95% confidence intervals (CIs) for the associations between the sixteen dietary categories and all-cause mortality. Risks are also presented per 10-point increase in the PDI indices stratified by quartiles of UPF consumption. Person-years were calculated from the date of study inclusion to the date of death or the end of follow-up, whichever came first. Confounders were considered in two separate models. Model 1 was cox-stratified for age and adjusted for sex and total energy intake. Model 2 was additionally adjusted for educational level, smoking status, physical activity level, and alcohol consumption. The proportional hazard assumption was checked using the Schoenfeld residuals test, but no evidence for violation of this assumption was found (all *P* > 0.05). Linear trends were tested by assigning median values to each quartile and entering this as continuous variables in the models. We evaluated additive interaction by estimating the relative excess risk due to interaction (RERI) based on continuous exposure variables [66, 67]. The hPDI was recoded into a risk factor by multiplying with − 1 for correct calculation of the RERI. A RERI of 0 means a lack of significant interaction on the additive scale.

To investigate associations between the dietary categories and daily GHGE and BWC, multiple linear regression models were used to calculate differences in adjusted mean GHGE and BWC with 95% CIs for the different dietary categories. Mean differences are also presented per 10-point increase in the PDI indices stratified by quartiles of UPF consumption. These analyses were adjusted for age, sex, and total energy intake (Model 1). Assumptions of the linear model, including multicollinearity, homoscedasticity, independence and normality of the residuals, and linearity of the association, were checked by calculating VIF-values (all < 2) and generating various plots (scale-location, Q-Q, and residuals vs. fitted values). Interaction on the additive scale was evaluated by testing the product term (β_3_) of the PDI indices and UPF consumption [66]. This estimate of interaction is not the same as RERI, but rather reflects the change in absolute values instead of a change in relative risks. A product term of 0 means a lack of significant additive interaction.

All statistical analyses were performed using R software (version 3.5.0). A two-sided *P*-value of < 0.05 was considered statistically significant.

## Results

### Baseline characteristics

Baseline characteristics, consumption of food groups and NOVA classes, and nutrient intakes are presented for quartiles of the PDI indices in Table 1. hPDI and uPDI scores ranged from a median of 47 points in the lowest quartile to 62 points in the highest quartile. Participants with higher scores of the hPDI were higher educated, were less likely to smoke, were more physically active, and had a lower BMI, whereas these trends were reversed for the uPDI. Besides, males tended to have higher scores of the uPDI and older participants more often had higher scores of the hPDI.

**Table 1:** Baseline characteristics, consumption of food groups and NOVA classes, and nutrient intakes according to quartiles of the hPDI and uPDI in the EPIC-NL cohort

Characteristics^a^	hPDI				uPDI			
	Q1 (≤ 50 points)	Q2 (50–54 points)	Q3 (54–58 points)	Q4 (> 58 points)	Q1 (≤ 49 points)	Q2 (49–54 points)	Q3 (54–58 points)	Q4 (> 58 points)
Participants (n)	10,319	8419	7837	8455	8978	9985	7438	8629
Females (%)	72.0	76.5	76.5	70.8	79.8	76.6	71.7	66.2
Age (years)	46.4 (13.0)	49.2 (11.9)	49.7 (11.4)	49.8 (11.0)	52.8 (9.4)	50.5 (10.9)	47.8 (11.9)	43.0 (13.3)
Educational level (%)								
Low	41.8	40.8	37.7	33.5	38.4	38.2	39.2	39.0
Medium	42.5	40.8	40.4	37.1	37.1	39.4	40.1	44.8
High	15.7	18.4	21.9	29.4	24.5	22.4	20.7	16.2
Smoking status (%)								
Never smoker	39.0	38.9	37.6	38.0	37.1	38.5	38.3	39.8
Former smoker	26.0	30.4	33.4	35.3	35.9	32.5	29.7	25.1
Current smoker	35.1	30.7	29.0	26.7	27.1	28.9	32.1	35.1
Physical activity level (%)								
Inactive	9.4	7.3	6.0	5.0	5.9	6.5	7.6	8.6
Moderately inactive	26.2	25.1	24.2	22.0	23.1	23.9	25.1	25.9
Moderately active	25.9	26.2	26.2	26.8	26.3	26.7	26.8	25.3
Active	38.5	41.4	43.6	46.3	44.8	42.9	40.5	40.2
BMI (kg/m^2^)	25.9 (4.2)	25.7 (3.9)	25.5 (3.9)	25.1 (3.7)	25.9 (3.9)	25.7 (3.9)	25.5 (3.9)	25.1 (4.0)
PDI scores								
hPDI	47 [45–49]	53 [52–54]	56 [55–57]	62 [60–64]	57 [53–61]	55 [51–59]	53 [49–57]	50 [46–54]
uPDI	58 [54–62]	54 [50–58]	53 [49–57]	50 [46–54]	47 [44–48]	52 [51–53]	56 [55–57]	62 [60–65]
NOVA classes (g/2000 kcal)								
MPF	2086 [1625–2667]	2288 [1800–2898]	2311 [1830–2901]	2256 [1781–2838]	2535 [2050–3142]	2343 [1885–2944]	2144 [1701–2704]	1840 [1456–2332]
PCI	20 [10–36]	16 [9–31]	16 [9–28]	15 [9–27]	16 [10–25]	16 [10–29]	17 [9–33]	20 [9–38]
PF	206 [154–302]	221 [170–308]	235 [182–325]	260 [201–365]	233 [181–323]	230 [177–324]	231 [170–334]	221 [166–320]
UPF	392 [299–514]	338 [262–441]	307 [242–396]	276 [217–353]	278 [219–353]	312 [243–404]	344 [267–443]	409 [308–537]
NOVA classes (%)								
MPF	76 [68–82]	79 [72–84]	80 [73–84]	79 [72–84]	82 [77–86]	80 [74–84]	77 [70–82]	72 [64–79]
PCI	0.7 [0.3–1.4]	0.5 [0.3–1.1]	0.5 [0.3–1.0]	0.5 [0.3–1.0]	0.5 [0.3–0.9]	0.5 [0.3–1.0]	0.6 [0.3–1.3]	0.8 [0.3–1.6]
PF	7.5 [5.1–11.6]	7.5 [5.3–11.4]	8.1 [5.7–11.8]	9.1 [6.4–13.7]	7.5 [5.4–11.1]	7.8 [5.5–11.7]	8.2 [5.6–12.8]	8.7 [5.9–13.5]
UPF	14 [10–19]	12 [8–16]	11 [8–14]	9.7 [7.0–13.1]	9.0 [6.7–12.0]	11 [8–14]	12 [9–16]	16 [11–21]
Dietary intake (g/2000 kcal)								
Healthy plant-based foods								
Vegetables	118 [87–157]	133 [99–177]	139 [103–186]	143 [105–190]	158 [119–208]	140 [105–184]	125 [94–165]	106 [78–141]
Fruit	124 [66–209]	167 [95–281]	194 [108–312]	222 [124–336]	233 [137–343]	188 [108–303]	153 [86–260]	113 [58–195]
Legumes	4.8 [1.6–10.1]	6.7 [2.7–12.9]	8.3 [3.6–15.2]	10 [5–18]	11 [5–18]	8.0 [3.6–14.8]	6.2 [2.5–12.3]	4.4 [1.3–9.4]
Whole grains	82 [39–116]	112 [78–143]	125 [95–157]	143 [112–174]	132 [103–161]	123 [91–155]	109 [72–145]	83 [35–123]
Nuts and seeds	4.4 [1.7–8.6]	5.5 [2.1–10.9]	6.7 [2.6–13.1]	9.2 [3.9–17.8]	7.1 [2.9–13.9]	6.2 [2.4–12.6]	5.7 [2.2–11.5]	5.1 [2.0–10.6]
Vegetable oils and fats	16 [10–23]	18 [12–26]	19 [13–27]	22 [14–29]	21 [14–28]	19 [12–27]	18 [11–26]	15 [9–23]
Tea and coffee	705 [481–979]	847 [604–1133]	884 [644–1173]	895 [668–1194]	955 [714–1244]	876 [646–1163]	794 [565–1071]	662 [442–923]
Unhealthy plant-based foods								
Refined grains	90 [58–134]	71 [45–106]	64 [41–95]	57 [37–82]	53 [35–76]	65 [41–93]	77 [50–113]	100 [65–146]
Potatoes	103 [69–146]	94 [61–134]	88 [56–129]	78 [50–119]	79 [52–117]	87 [57–128]	95 [62–140]	106 [70–148]
Juices	72 [23–150]	53 [13–133]	44 [9–120]	27 [4–88]	31 [5–97]	48 [12–127]	54 [13–135]	65 [18–146]
(Sugar) sweetened beverages	125 [49–238]	77 [20–168]	53 [12–132]	31 [4–84]	31 [4–83]	57 [13–135]	83 [22–175]	141 [57–261]
Sweets and desserts	56 [36–78]	54 [34–77]	53 [34–76]	51 [33–73]	42 [27–60]	51 [33–71]	58 [38–81]	69 [47–93]
Animal-based foods								
Meat	119 [88–149]	107 [75–138]	99 [67–131]	86 [54–117]	110 [76–142]	104 [70–136]	102 [70–134]	99 [68–131]
Animal fats	3.1 [1.6–7.3]	2.7 [1.6–5.5]	2.6 [1.5–4.6]	2.6 [1.6–4.1]	3.3 [2.0–6.9]	2.9 [1.7–5.6]	2.6 [1.5–4.9]	2.1 [1.2–3.7]
Eggs	17 [11–26]	15 [8–22]	12 [7–20]	9.6 [5.2–15.7]	17 [11–25]	14 [8–22]	12 [7–20]	9.9 [5.1–16.7]
Fish and seafood	9.7 [4.2–16.9]	7.9 [3.2–15.2]	6.7 [2.7–14.0]	4.9 [1.8–11.0]	11 [5–18]	8.4 [3.5–15.6]	6.2 [2.5–12.9]	4.1 [1.4–9.3]
Dairy products	384 [218–579]	410 [240–602]	401 [242–587]	380 [226–564]	474 [315–655]	419 [253–603]	371 [216–552]	295 [166–478]
Miscellaneous^b^	88 [53–139]	75 [44–124]	64 [38–106]	52 [31–88]	88 [51–141]	70 [41–117]	64 [38–109]	58 [35–96]
Nutrient intake								
Energy (kcal/day)	2010 (610)	2000 (586)	2049 (590)	2213 (634)	2061 (579)	2014 (587)	2049 (621)	2142 (656)
Ethanol (g/day)	10.4 (15.0)	10.3 (14.3)	11.2 (15.4)	12.8 (16.6)	11.7 (14.2)	11.4 (15.1)	11.3 (16.1)	10.1 (16.1)
Fiber (g/day)	20.3 (5.6)	22.8 (5.6)	24.8 (5.8)	28.2 (6.6)	26.2 (6.3)	24.2 (6.2)	23.1 (6.5)	21.6 (6.5)
Protein (E%)	15.4 (2.4)	15.6 (2.4)	15.5 (2.3)	15.2 (2.2)	16.6 (2.2)	15.7 (2.2)	15.1 (2.1)	14.1 (2.2)
Plant protein (E%)	5.1 (0.9)	5.5 (0.9)	5.7 (1.0)	6.1 (1.0)	5.7 (1.0)	5.6 (1.0)	5.5 (1.0)	5.4 (1.0)
Animal protein (E%)	10.3 (2.5)	10.1 (2.5)	9.8 (2.5)	9.1 (2.5)	10.9 (2.5)	10.1 (2.5)	9.6 (2.4)	8.7 (2.3)
Saturated fatty acids (E%)	14.2 (2.5)	14.1 (2.6)	13.9 (2.5)	13.8 (2.5)	14.3 (2.5)	14.1 (2.6)	13.9 (2.5)	13.7 (2.5)
Sodium (mg/day)	2338 (829)	2356 (814)	2388 (816)	2558 (862)	2554 (825)	2375 (803)	2342 (837)	2345 (862)
Sugar (E%)	23.2 (6.1)	23.0 (6.1)	22.8 (5.9)	22.4 (6.0)	21.7 (5.3)	22.5 (5.9)	23.1 (6.2)	24.3 (6.4)

*hPDI* healthful plant-based diet index, *MPF* unprocessed/minimally processed foods, *PCI* processed culinary ingredients, *PF* processed foods, *Q* quartile, *SD* standard deviation, *uPDI* unhealthful plant-based diet index, *UPF* ultra-processed foods

^a^Continuous, normally distributed variables are presented as mean (SD), continuous, not-normally distributed variables are presented as median with interquartile range [25–75th percentile], and categorical variables are presented as percentage

^b^Mixed dishes that contain substantial amounts of animal sourced ingredients e.g. soups, pizza, and mayonnaise. Supplemental Table 1 displays detailed information on included food items

Participants with higher uPDI scores consumed almost 50% more UPF (Q1vsQ4: 278 to 409 g/2000 kcal) compared to those with lower scores (Table 1). These differences in UPF consumption were mainly due to a higher consumption of ultra-processed (sugar) sweetened beverages (Q1vsQ4: 31 to 141 g/2000 kcal), sweets and desserts (Q1vsQ4: 32 to 48 g/2000 kcal), refined grains (Q1vsQ4: 20 to 24 g/2000 kcal), and juices (Q1vsQ4: 3.1 to 6.4 g/2000 kcal), although the consumption of ultra-processed vegetable oils and fats (Q1vsQ4: 16 to 12 g/2000 kcal) was lower (Supplemental Table 3). Reversed associations were observed for the hPDI with the consumption of total and specific UPF (Table 1, Supplemental Table 4). Exceptions were ultra-processed sweets and desserts as well as meat for which the consumption was similar and lower across quartiles, respectively.

Whilst participants with higher scores of the hPDI had higher intakes of fiber, sodium, and alcohol and a lower intake of sugar, reversed associations were observed for quartiles of the uPDI (Table 1). Protein intake was similar across quartiles of the hPDI, but lower for participants with higher uPDI scores. For both the hPDI and uPDI, intakes of energy and saturated fatty acids were slightly higher and lower, respectively, for participants with higher scores compared to those with lower scores.

### Contribution of UPF consumption to environmental impact

**Fig. 1:**
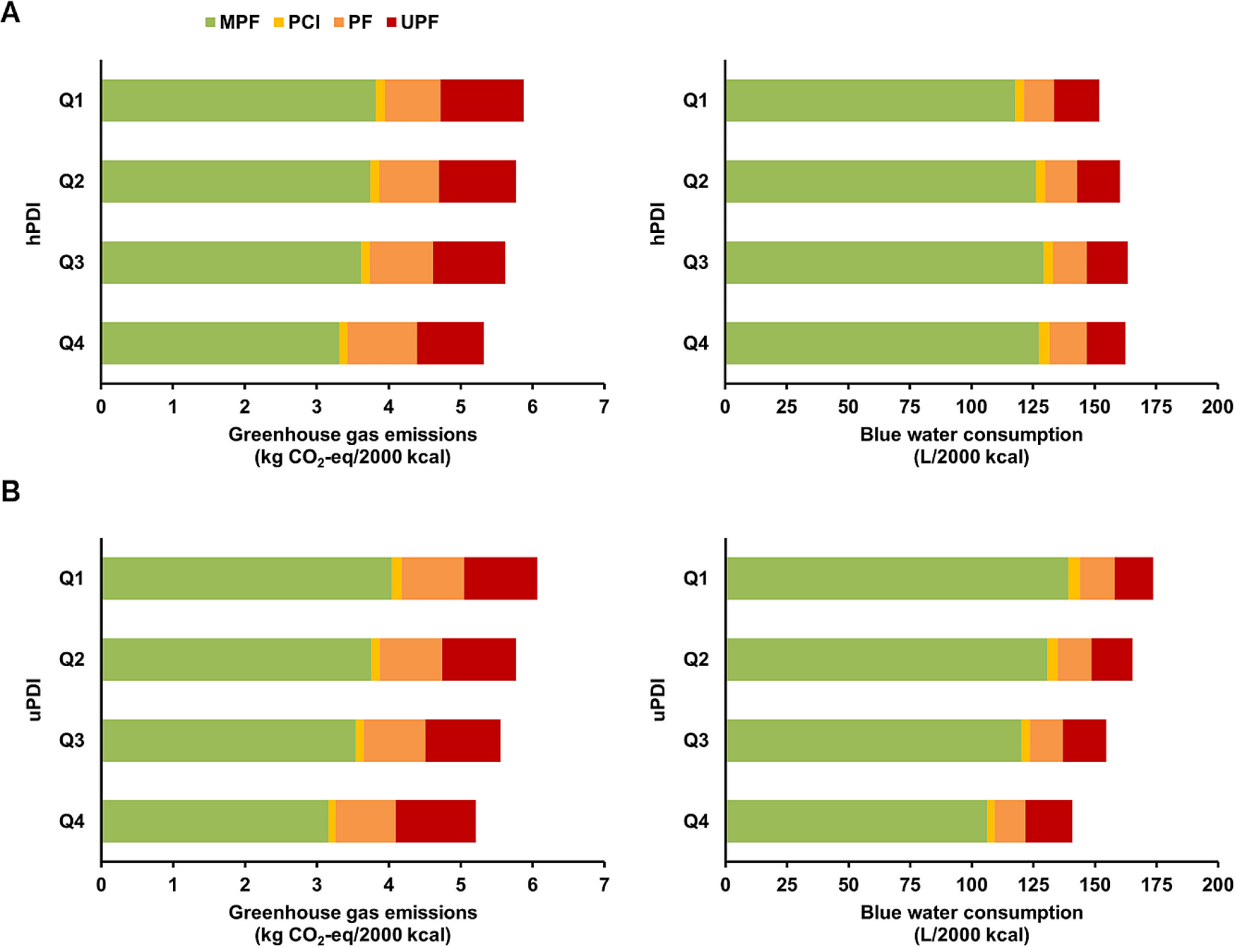
Contribution of NOVA classes to diet-related greenhouse gas emissions and blue water consumption according to quartiles of the (**A**) hPDI and (**B**) uPDI in the EPIC-NL cohort. Q1 represents lower scores, whereas Q4 represents higher scores. *hPDI* healthful plant-based diet index, *MPF* unprocessed/minimally processed foods, *PCI* processed culinary ingredients, *PF* processed foods, *Q* quartile, uPDI unhealthful plant-based diet index, *UPF* ultra-processed foods

Following UPF consumption patterns, UPF-related environmental impacts varied between quartiles of the hPDI and uPDI (Fig. 1, Supplemental Tables 3 and 4). UPF contributed 21% to GHGE and 14% to BWC for diets of participants with higher scores of the uPDI compared to 17% and 9%, respectively, for lower scores. These differences in UPF-related environmental impacts were mostly due to an increase in the consumption of sweets and desserts, (sugar) sweetened beverages, juices, and refined grains. Again, opposite trends were found for the hPDI, although these were less pronounced. Across quartiles of the hPDI and uPDI, UPF-related environmental impacts were largely coming from ultra-processed meat (GHGE: 6.5–7.8%, BWC: 1.9–2.7%), sweets and desserts (GHGE: 1.8–3.4%, BWC: 2.1–4.3%), and dairy products (GHGE: 2.3–2.9%, BWC: 0.7–0.9%).

### Interaction between PDI indices and UPF consumption: all-cause mortality

**Fig. 2:**
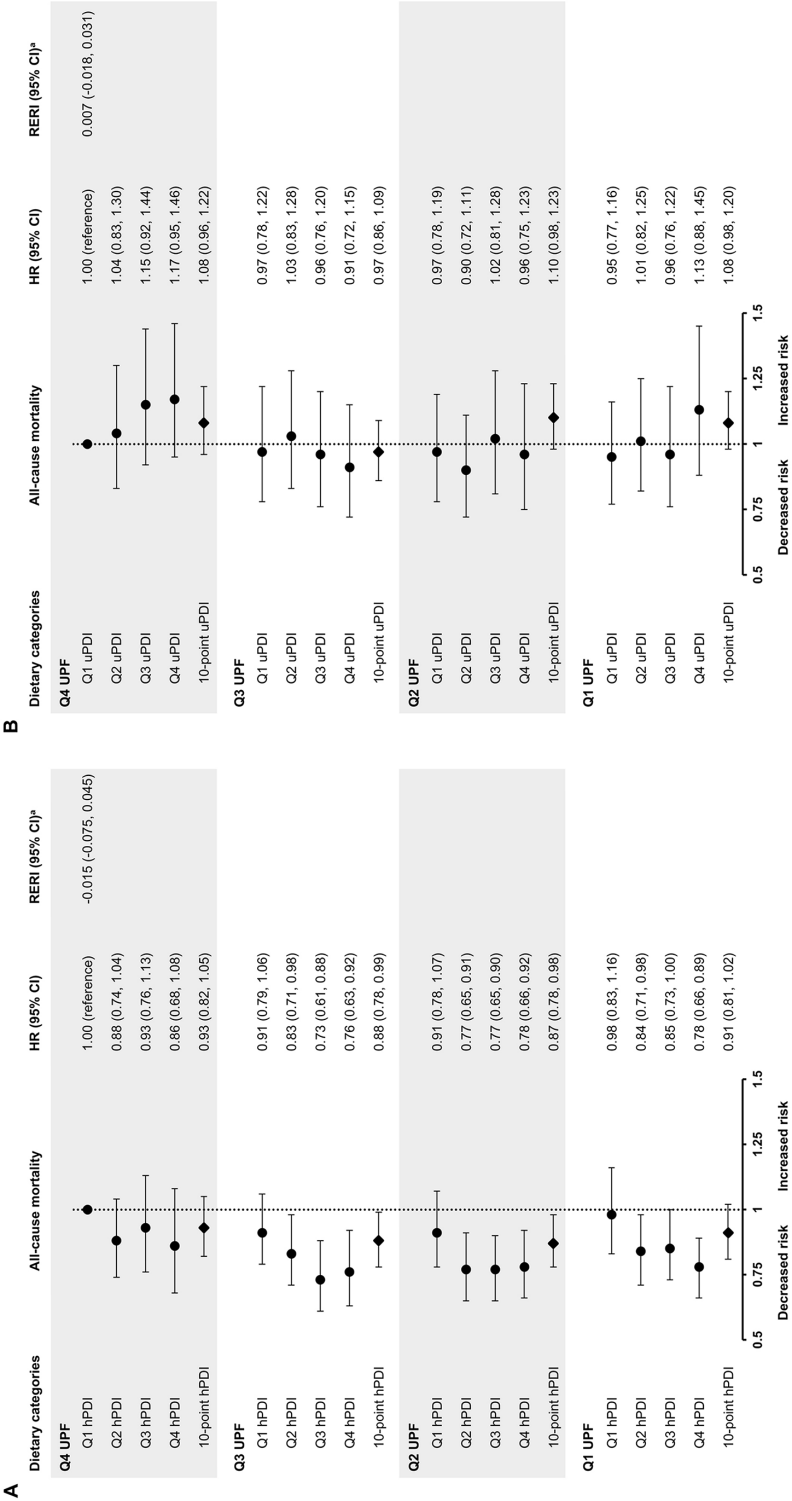
Associations between the (**A**) hPDI and (**B**) uPDI and all-cause mortality risk by UPF consumption in the EPIC-NL cohort. HRs are adjusted for age, sex, total energy intake, educational level, smoking status, physical activity level, and alcohol consumption. Q1 represents lower scores or consumption, whereas Q4 represents higher scores or consumption. ^a^Relative excess all-cause mortality risk due to interaction between a 10-point decrease in the hPDI score or 10-point increase in the uPDI score and a 100 g/2000 kcal increase in UPF consumption. The hPDI was recoded into a risk factor for correct calculation of the RERI. *CI* confidence interval, *HR* hazard ratio, *hPDI* healthful plant-based diet index, *Q* quartile, *uPDI* unhealthful plant-based diet index, *UPF* ultra-processed foods

Compared to participants with a lower hPDI score and higher UPF consumption (Q1hPDI/Q4UPF), those with both a higher hPDI score and lower UPF consumption (Q4hPDI/Q1UPF) were associated with a statistically significant 22% (HR: 0.78, 95% CI: 0.66, 0.89) decreased all-cause mortality risk (Fig. 2, Supplemental Table 5). Favorable associations (HR: 0.86, 95% CI: 0.68, 1.08) were observed for a solely higher hPDI score (Q4hPDI/Q4UPF), whereas we found no differences (HR: 0.98, 95% CI: 0.83, 1.16) in mortality risk for a solely lower UPF consumption (Q1hPDI/Q1UPF). Somewhat reversed trends were observed for the uPDI, although not statistically significant. Respective HRs for participants with a solely lower UPF consumption (Q1uPDI/Q1UPF), a solely higher uPDI score (Q4uPDI/Q4UPF), and both a higher uPDI score and lower UPF consumption (Q4uPDI/Q1UPF) were 0.95 (95% CI: 0.77, 1.16), 1.17 (95% CI: 0.95, 1.46), and 1.13 (95% CI: 0.88, 1.45).

Overall, there was no relative excess risk due to interaction between the hPDI (RERI: -0.015, 95% CI: -0.075, 0.045) or uPDI (RERI: 0.007, 95% CI: -0.018, 0.031) and UPF consumption in the associations with all-cause mortality, indicating that the combined associations of adherence to a healthful or unhealthful plant-based diet and amount of UPF consumed were additive (Fig. 2, Supplemental Table 5). Despite this, a 10-point increase in scores of the hPDI was associated with a statistically significant 13% (HR: 0.87, 95% CI: 0.78, 0.98) and 12% (HR: 0.88, 95% CI: 0.78, 0.99) decreased mortality risk only in the second and third quartiles of UPF consumption, respectively. Associations were not statistically significant for the uPDI.

### Interaction between PDI indices and UPF consumption: environmental impact

Compared to diets of participants with a lower hPDI score and higher UPF consumption (Q1hPDI/Q4UPF), diet-related GHGE were 6.8% (95% CI: -7.4, -6.1) lower and BWC was 13.1% (95% CI: 11.6, 14.8) higher for both a higher hPDI score and lower UPF consumption (Q4hPDI/Q1UPF) (Tables 2 and 3). For a solely lower UPF consumption (Q1hPDI/Q1UPF) and a solely higher hPDI score (Q4hPDI/Q4UPF), respectively, these estimates were 1.4% (95% CI: 0.3, 2.4) and − 7.4% (95% CI: -8.6, -6.4) for GHGE and 1.6% (95% CI: -0.5, 3.7) and 9.6% (95% CI: 7.2, 12.0) for BWC. Different patterns were observed for the uPDI; GHGE were lower for diets of participants containing lower amounts of UPF and BWC was lower for participants with a higher adherence to an unhealthful plant-based diet. Compared to the Q1uPDI/Q4UPF category, mean differences in BWC were 1.6% (95% CI: -0.5, 3.7), -15.4% (95% CI: -17.4, -13.3), and − 15.4% (95% CI: -18.0, -12.8) for a solely lower UPF consumption (Q1uPDI/Q1UPF), a solely higher uPDI score (Q4uPDI/Q4UPF), and both a higher uPDI score and lower UPF consumption (Q4uPDI/Q1UPF), respectively.

**Table 2:** Associations between the PDI indices and diet-related GHGE (kg CO_2_-eq/day) by UPF consumption in the EPIC-NL cohort

		hPDI										*P* value for trend^a^	Measure of additive interaction	
		Q1 (≤ 50 points)		Q2 (50–54 points)		Q3 (54–58 points)		Q4 (> 58 points)		Per 10-point increase				
		(∆)Mean	95% CI	∆Mean	95% CI	∆Mean	95% CI	∆Mean	95% CI	∆Mean	95% CI		β_3_^b^	95% CI
UPF	Q4 (> 433 g/2000 kcal)	5.92^c^ 100%	Reference	-0.16 -2.7%	-0.21, -0.11 -3.5, -1.9	-0.24 -4.1%	-0.29, -0.18 -4.9, -3.0	-0.44 -7.4%	-0.51, -0.38 -8.6, -6.4	-0.26	-0.30, -0.23	< 0.001	0.0000	0.0000, 0.0001 *P* = 0.316
	Q3 (328–433 g/2000 kcal)	-0.01 -0.2%	-0.06, 0.03 -1.0, 0.5	-0.16 -2.7%	-0.21, -0.11 -3.5, -1.9	-0.28 -4.7%	-0.33, -0.23 -5.6, -3.9	-0.52 -8.8%	-0.58, -0.47 -9.8, -7.9	-0.34	-0.37, -0.30	< 0.001		
	Q2 (251–328 g/2000 kcal)	0.07 1.2%	0.01, 0.12 0.2, 2.0	-0.13 -2.2%	-0.18, -0.08 -3.0, -1.4	-0.27 -4.6%	-0.32, -0.22 -5.4, -3.7	-0.50 -8.4%	-0.55, -0.46 -9.3, -7.8	-0.38	-0.41, -0.35	< 0.001		
	Q1 (≤ 251 g/2000 kcal)	0.08 1.4%	0.02, 0.14 0.3, 2.4	-0.01 -0.2%	-0.06, 0.04 -1.0, 0.7	-0.17 -2.9%	-0.22, -0.12 -3.7, -2.0	-0.40 -6.8%	-0.44, -0.36 -7.4, -6.1	-0.38	-0.41, -0.34	< 0.001		

		uPDI										*P* value for trend^a^	Measure of additive interaction	
		Q1 (≤ 50 points)		Q2 (50–54 points)		Q3 (54–58 points)		Q4 (> 58 points)		Per 10-point increase				
		(∆)Mean	95% CI	∆Mean	95% CI	∆Mean	95% CI	∆Mean	95% CI	∆Mean	95% CI		β_3_^b^	95% CI
UPF	Q4 (> 433 g/2000 kcal)	6.42^c^ 100%	Reference	-0.33 -5.1%	-0.40, -0.26 -6.2, -4.0	-0.60 -9.3%	-0.67, -0.53 -10.4, -8.3	-0.98 -15.3%	-1.04, -0.91 -16.2, -14.2	-0.61	-0.64, -0.57	< 0.001	-0.0002	-0.0003, -0.0001 *P* < 0.001
	Q3 (328–433 g/2000 kcal)	-0.23 -3.6%	-0.30, -0.16 -4.7, -2.5	-0.60 -9.3%	-0.67, -0.53 -10.4, -8.3	-0.78 -12.1%	-0.85, -0.71 -13.2, -11.1	-1.09 -17.0%	-1.15, -1.02 -17.9, -15.9	-0.53	-0.56, -0.50	< 0.001		
	Q2 (251–328 g/2000 kcal)	-0.33 -5.1%	-0.40, -0.27 -6.2, -4.2	-0.69 -10.7%	-0.75, -0.62 -11.7, -9.7	-0.89 -13.9%	-0.96, -0.82 -15.0, -12.8	-1.11 -17.3%	-1.18, -1.03 -18.4, -16.0	-0.49	-0.52, -0.46	< 0.001		
	Q1 (≤ 251 g/2000 kcal)	-0.33 -5.1%	-0.39, -0.27 -6.1, -4.2	-0.71 -11.1%	-0.77, -0.64 -12.0, -10.0	-0.86 -13.4%	-0.94, -0.79 -14.6, -12.3	-1.22 -19.0%	-1.30, -1.14 -20.2, -17.8	-0.51	-0.54, -0.48	< 0.001		

*CI* confidence interval, *GHGE* greenhouse gas emissions, *hPDI* healthful plant-based diet index, *Q* quartile, *uPDI* unhealthful plant-based diet index, *UPF* ultra-processed foods

(∆)Means are adjusted for age, sex, and total energy intake

^a^Linear trend test was conducted by assigning median values to each quartile and entering this as continuous variables in the models

^b^Absolute excess GHGE due to interaction between a 10-point increase in the hPDI or uPDI score and a 100 g/2000 kcal decrease in UPF consumption

^c^Represents the mean value in the reference category (Q1hPDI/Q4UPF or Q1uPDI/Q4UPF)

We observed a statistically significant negative and positive additive interaction between the uPDI and UPF consumption in the associations with GHGE (β_3_: -0.0002, 95% CI: -0.0003, -0.0001) and BWC (β_3_: 0.005, 95% CI: 0.001, 0.010), respectively (Tables 2 and 3). The combined associations of a higher adherence to an unhealthful plant-based diet and lower consumption of UPF with GHGE were less than additive, whereas these associations were more than additive for BWC. Within the highest quartile of UPF consumption, GHGE decreased with 0.61 kg CO_2_-eq/day per 10-point increase in the uPDI compared to a 0.49–0.53 kg CO_2_-eq/day decrease in the lowest three quartiles. BWC decreased with 17.0 L/day per 10-point increase in the uPDI in the lowest quartile of UPF consumption compared to a 11.5–14.1 L/day decrease in the highest three quartiles. In contrast, a borderline statistically significant negative additive interaction was found between the hPDI and UPF consumption in the associations with BWC (β_3_: -0.005, 95% CI: -0.009, 0.000), meaning that the combined associations of a higher adherence to a healthful plant-based diet and lower consumption of UPF with BWC were less than additive. Within the lowest quartile of UPF consumption, BWC of the diet increased with 10.9 L/day per 10-point increase in the hPDI, whereas this increase was 8.0–8.4 L/day in the highest three quartiles.

**Table 3:** Associations between the PDI indices and diet-related BWC (L/day) by UPF consumption in the EPIC-NL cohort

		hPDI										*P* value for trend^a^	Measure of additive interaction	
		Q1 (≤ 50 points)		Q2 (50–54 points)		Q3 (54–58 points)		Q4 (> 58 points)		Per 10-point increase				
		(∆)Mean	95% CI	∆Mean	95% CI	∆Mean	95% CI	∆Mean	95% CI	∆Mean	95% CI		β_3_^b^	95% CI
UPF	Q4 (> 433 g/2000 kcal)	141.5^c^ 100%	Reference	7.2 5.1%	4.7, 9.6 3.3, 6.8	9.5 6.7%	6.6, 12.4 4.7, 8.8	13.6 9.6%	10.2, 17.0 7.2, 12.0	8.4	6.7, 10.1	< 0.001	-0.005	-0.009, 0.000 *P* = 0.055
	Q3 (328–433 g/2000 kcal)	1.6 1.1%	-0.7, 3.9 -0.5, 2.8	7.7 5.4%	5.2, 10.1 3.7, 7.1	10.1 7.1%	7.5, 12.7 5.3, 9.0	14.8 10.5%	12.1, 17.5 8.6, 12.4	8.0	6.4, 9.6	< 0.001		
	Q2 (251–328 g/2000 kcal)	2.5 1.8%	-0.1, 5.1 0.1, 3.6	3.9 2.8%	1.4, 6.5 1.0, 4.6	10.4 7.3%	7.9, 12.9 5.6, 9.1	14.9 10.5%	12.4, 17.3 8.8, 12.2	8.1	6.5, 9.8	< 0.001		
	Q1 (≤ 251 g/2000 kcal)	2.2 1.6%	-0.7, 5.2 -0.5, 3.7	6.5 4.6%	3.9, 9.2 2.8, 6.5	11.1 7.8%	8.6, 13.6 6.1, 9.6	18.6 13.1%	16.4, 20.9 11.6, 14.8	10.9	9.1, 12.7	< 0.001		

		uPDI										*P* value for trend^a^	Measure of additive interaction	
		Q1 (≤ 50 points)		Q2 (50–54 points)		Q3 (54–58 points)		Q4 (> 58 points)		Per 10-point increase				
		(∆)Mean	95% CI	∆Mean	95% CI	∆Mean	95% CI	∆Mean	95% CI	∆Mean	95% CI		β_3_^b^	95% CI
UPF	Q4 (> 433 g/2000 kcal)	161.5^c^ 100%	Reference	-6.3 -3.9%	-10.0, -2.7 -6.2, -1.7	-15.1 -9.3%	-18.8, -11.5 -11.6, -7.1	-24.8 -15.4%	-28.1, -21.4 -17.4, -13.3	-13.5	-15.1, -11.8	< 0.001	0.005	0.001, 0.010 *P* = 0.012
	Q3 (328–433 g/2000 kcal)	0.3 0.2%	-3.3, 4.0 -2.0, 2.5	-8.8 -5.4%	-12.3, -5.3 -7.6, -3.3	-16.3 -10.1%	-19.9, -12.7 -12.3, -7.9	-22.6 -14.0%	-26.2, -19.0 -16.2, -11.8	-14.1	-15.6, -12.5	< 0.001		
	Q2 (251–328 g/2000 kcal)	-3.7 -2.3%	-7.2, -0.2 -4.5, -0.1	-10.7 -6.6%	-14.2, -7.2 -8.8, -4.5	-15.1 -9.3%	-18.8, -11.4 -11.6, -7.1	-21.3 -13.2%	-25.1, -17.4 -15.5, -10.8	-11.5	-13.1, -9.8	< 0.001		
	Q1 (≤ 251 g/2000 kcal)	2.6 1.6%	-0.8, 6.0 -0.5, 3.7	-8.3 -5.1%	-11.8, -4.8 -7.3, -3.0	-17.9 -11.1%	-21.7, -14.1 -13.4, -8.7	-24.9 -15.4%	-29.0, -20.7 -18.0, -12.8	-17.0	-18.8, -15.2	< 0.001		

*BWC* blue water consumption, *CI* confidence interval, *hPDI* healthful plant-based diet index, *Q* quartile, *uPDI* unhealthful plant-based diet index, *UPF* ultra-processed foods

(∆)Means are adjusted for age, sex, and total energy intake

^a^Linear trend test was conducted by assigning median values to each quartile and entering this as continuous variables in the models

^b^Absolute excess BWC due to interaction between a 10-point increase in the hPDI or uPDI score and a 100 g/2000 kcal decrease in UPF consumption

^c^Represents the mean value in the reference category (Q1hPDI/Q4UPF or Q1uPDI/Q4UPF)

## Discussion

In this prospective study, we explored the consumption of UPF in healthful and unhealthful plant-based diets (according to the hPDI and uPDI), reported in the nineties, and examined their associations with all-cause mortality, GHGE, and BWC. We observed lower mortality risks for a greater adherence to a healthful plant-based diet, which were comparable between lower and higher consumers of UPF. There was no consistent evidence for the combined associations of unhealthful plant-based diets and UPF consumption with risk of all-cause mortality. Besides, we identified discrepancies between indicators of environmental impact for healthful plant-based diets, but not for unhealthful plant-based diets. Diets higher in healthy plant-based foods were associated with lower GHGE and a higher BWC, whereas both indicators were lower for diets higher in unhealthy plant-based foods. Modest negative and positive additive interactions were found between the uPDI and UPF consumption, indicating that the benefits of more unhealthful plant-based diets on GHGE and BWC were larger among high and low consumers of UPF, respectively.

Although limited to two studies, plant-based diets and UPF consumption have been associated before [45, 49]. A cross-sectional analysis performed in a sample of 21,212 French adults found that a higher adherence to a plant-based diet was associated with a higher consumption of UPF, with an at least 6% higher consumption among vegans compared to meat-eaters [45]. Another French study that included 1,774 adults revealed that such a higher UPF consumption was mostly associated with unhealthful plant-based diets, containing high amounts of sweet beverages, breakfast cereals, savory pastries, confectionary and chocolate, and fruit juices [49]. In line with Salomé et al. [49], we observed an almost 50% higher consumption of UPF in participants with a higher adherence to an unhealthful plant-based diet compared to those with a lower adherence (when comparing extreme quartiles). More unhealthful plant-based diets were characterized by a higher consumption of ultra-processed (sugar) sweetened beverages, juices, sweets and desserts, and refined grains.

Building upon these earlier findings, our study is the first longitudinal study to investigate all-cause mortality risk of UPF consumption in plant-based diets. The protective role of plant-based diets for human health has been highlighted in multiple meta-analyses [18, 68, 69] and higher proportions of UPF in the diet have repeatedly been linked to various negative health effects [40–42]. In the present study, we observed a 12–13% decreased risk of all-cause mortality per 10-point increase in the hPDI in the second and third quartiles of UPF consumption, which is similar to the 10–11% decreases reported by previous studies [30, 31, 33]. Interestingly, there were no significant differences in mortality risk between higher and lower UPF consumption among participants with both lower and higher hPDI scores. These results are not in line with earlier prospective cohort studies that reported 14–62% increased risks of all-cause mortality with higher shares of UPF in the diet (without considering adherence to a plant-based diet) [70–75]. A possible explanation could be that the difference in UPF consumption between higher and lower UPF diets may have been insufficient in our study population to detect a significant association. For instance, other studies in which extreme quartiles were compared, reported an at least four times higher UPF consumption, whereas we only measured a two times higher consumption across quartiles [70–73]. Furthermore, UPF consumption in our study population (12% of food weight) was lower than a 20-year follow-up in a sub-sample of the EPIC-NL cohort (18% of food weight) [76], and not comparable to the higher amounts reported in more recent dietary surveys in Western countries (24–60% of energy intake) [48, 77, 78]. As such, the amount of UPF in the diet may have been too low to adversely influence mortality risk. Since UPF and MPF were found to be inversely associated across quartiles of healthful and unhealthful plant-based diets, it could additionally be argued whether our results should be ascribed to the lower levels of MPF consumed instead of the higher levels of UPF.

The potential negative environmental impacts of UPF have been described in multiple reviews [43, 44, 79], with special concern for the impacts of the growing amount of highly processed plant-based meat and dairy alternatives on the market [80]. In the current study, we found that UPF-related environmental impacts differed across levels of healthful and unhealthful plant-based diets; UPF contributed less to GHGE and BWC for more healthful plant-based diets, whereas contributions were higher for more unhealthful plant-based diets. In line with Vellinga et al. [63], who quantified the environmental impacts of diets with a higher share of UPF and ultra-processed drinks (UPD) separately, these variations in UPF-related environmental impacts mainly came from UPD, such as ultra-processed (sugar) sweetened beverages and juices. Nonetheless, UPF contributed considerably less to total environmental impacts than MPF; GHGE were largely related to the consumption of meat and dairy products, while BWC was mostly driven by the consumption of fruit, tea and coffee, juices, and meat. In fact, the amount of plant-based or animal-based foods in the diet explained considerably more of the differences in environmental impacts than the consumption of UPF. These findings are in line with earlier conclusions that plant-based UPF, including sweets and desserts, snacks, and soft drinks, produce less GHGE than conventional meat and dairy products [43]. Large reductions in GHGE could thus possibly be achieved when switching to more plant-based diets and limiting the consumption of animal-based foods, irrespective of UPF consumption.

It should be emphasized, however, that different dimensions of diet-related environmental impacts may not be compatible with each other and could lead to contrasting conclusions. While diets rich in plant-based foods have consistently shown to be associated with lower GHGE and land use than conventional diets, high amounts of blue water are needed for the irrigation of specific plant foods, such as nuts from California and oranges from Spain [15, 16]. Indeed, we observed a higher BWC among participants consuming a diet higher in healthy plant-based foods compared to those consuming lower amounts, whereas BWC was lower for more unhealthful plant-based diets rich in UPF. A recent study that investigated the trade-offs between GHGE and BWC in optimizing more sustainable (plant-based) diets, concluded there is good alignment between these indicators for high emitting foods but some diversity for low emitting foods [81]. Especially unprocessed plant-based foods, such as nuts and seeds, fruit, and vegetables, are major contributors to the total blue water footprint [26, 27]. The production of these crops could be of concern in regions with water scarcity where groundwater sources are depleted and water irrigation is limited [26]. Therefore, switching to more water-efficient crops, such as apples instead of citrus fruits, and/or improving water management could prevent large-scale depletion of blue water resources [62, 82]. Considering that unhealthful plant-based diets were lower in GHGE and BWC, but may adversely influence health, clear dietary recommendations on the types and amounts of plant-based foods to incorporate in the diet are needed to maintain or even improve nutritional status within (environmentally) sustainable food systems.

### Strengths and limitations

Our study is at the root of the major dietary problems that the Western world faces at the moment. While shifting towards more plant-based diets is gaining more attention from both professionals and consumers, the increasing consumption of plant-based UPF may go at the expense of the health and environmental benefits of these diets. As such, the present study adds to the existing literature on plant-based diets and UPF by providing insights into the trade-offs between health and environmental impacts. Strengths of this study include the use of a large nationally representative cohort of the Dutch population with validated measures and long mean follow-up time of 17.5 years.

Nonetheless, some limitations need to be addressed. First, usual dietary intake was self-reported using an FFQ which is prone to measurement errors and may not reflect actual intake, leading to misclassification of dietary categories [83]. Besides, the FFQ used in the present study was not designed to collect data specifically on food processing or plant-based foods. Although each food item was classified into the most likely NOVA class or PDI category, misclassification of some foods cannot be ruled out. Second, since we only used baseline dietary information from the nineties, we were not able to estimate dietary changes over time or include newer plant-based UPF such as meat replacers and dairy substitutes in our analyses. Considering that modern food production and consumption have shifted to include more (plant-based) UPF, it is important to replicate our findings using more recent dietary intake information with repeated measurements to better understand the role of UPF in plant-based diets with health and environmental outcomes [5, 46, 47]. Third, the NOVA classification has been criticized before due to its very broad definition of the UPF category that includes markedly differing foods [84]. A recent study reported that distinct UPF consumption patterns (cold/warm savory snack pattern, sweet snack pattern, or traditional Dutch cuisine pattern) were differently associated with incident type 2 diabetes, suggesting that not all UPF can be considered equally harmful to our health [85]. However, no better alternative than the NOVA classification has been proposed to date and it is the most used and recommended approach for classifying UPF that enables comparison in public health nutrition [86, 87]. Fourth, the indicators for environmental impact used in the present study are based on Dutch production and consumption practices and may therefore not apply to other countries where life cycles of food products could differ.

## Conclusions

UPF consumption was considerably lower and higher for participants consuming more healthy and unhealthy plant-based foods, respectively. Despite this, the amount of plant-based foods in the diet seemed to explain more of the observed differences in all-cause mortality risk and environmental impacts than UPF consumption. While a greater adherence to a healthful plant-based diet was associated with lower mortality risks, lower GHGE, and a higher BWC, no apparent conflict between GHGE and BWC was identified for more unhealthful plant-based diets. Thus, based on our study population, replacing animal products with healthy plant-based foods in the diet could improve human health and reduce most aspects of environmental impact (e.g. GHGE) irrespective of UPF consumption, although special concern needs to be given to the high BWC of specific plant foods. Future research should clarify whether this conclusion is generalizable to current settings in which populations consume higher amounts and different types of (plant-based) UPF.

## Electronic supplementary material

Below is the link to the electronic supplementary material.

Supplementary Material 1

## Data Availability

Internal rules apply to the use of EPIC-NL data. Requests to work with the data are dependent on approval and should be sent to the cohort staff.

## References

[1] FAO, IFAD, UNICEF, WFP, WHO (2018) The State of Food Security and Nutrition in the World 2018. Building climate resilience for food security and nutrition. FAO, Rome

[2] Tilman D, Clark M (2014) Global diets link environmental sustainability and human health. Nature 515:518–522. 10.1038/NATURE13959 25383533 10.1038/nature13959

[3] GBD 2017 Diet Collaborators (2019) Health effects of dietary risks in 195 countries, 1990–2017: a systematic analysis for the global burden of Disease Study 2017. Lancet 393:1958–1972. 10.1016/S0140-6736(19)30041-8 30954305 10.1016/S0140-6736(19)30041-8 PMC6899507

[4] WHO (2003) Diet, nutrition and the prevention of chronic diseases. Report of a joint WHO/FAO expert consultation. WHO Technical Report Series, Geneve

[5] Zobel EH, Hansen TW, Rossing P, von Scholten BJ (2016) Global changes in Food Supply and the obesity epidemic. Curr Obes Rep 5:449–455. 10.1007/S13679-016-0233-8 27696237 10.1007/s13679-016-0233-8

[6] Abete I, Romaguera D, Vieira AR et al (2014) Association between total, processed, red and white meat consumption and all-cause, CVD and IHD mortality: a meta-analysis of cohort studies. Br J Nutr 112:762–775. 10.1017/S000711451400124X 24932617 10.1017/S000711451400124X

[7] Farvid MS, Sidahmed E, Spence ND et al (2021) Consumption of red meat and processed meat and cancer incidence: a systematic review and meta-analysis of prospective studies. Eur J Epidemiol 36:937–951. 10.1007/S10654-021-00741-9 34455534 10.1007/s10654-021-00741-9

[8] Larsson SC, Orsini N (2014) Red meat and processed meat consumption and all-cause mortality: a Meta-analysis. Am J Epidemiol 179:282–289. 10.1093/AJE/KWT261 24148709 10.1093/aje/kwt261

[9] Wang X, Lin X, Ouyang YY et al (2016) Red and processed meat consumption and mortality: dose–response meta-analysis of prospective cohort studies. Public Health Nutr 19:893–905. 10.1017/S1368980015002062 26143683 10.1017/S1368980015002062 PMC10270853

[10] Zhang R, Fu J, Moore JB et al (2021) Processed and unprocessed red meat consumption and risk for type 2 diabetes Mellitus: an updated Meta-analysis of Cohort studies. Int J Environ Res Public Health 18:10788. 10.3390/ijerph182010788 34682532 10.3390/ijerph182010788 PMC8536052

[11] Foley JA, Defries R, Asner GP et al (2005) Global consequences of land use. Science 309:570–574. 10.1126/SCIENCE.1111772 16040698 10.1126/science.1111772

[12] Garnett T (2013) Food sustainability: problems, perspectives and solutions. Proc Nutr Soc 72:29–39. 10.1017/S0029665112002947 23336559 10.1017/S0029665112002947

[13] Steffen W, Richardson K, Rockström J et al (2015) Planetary boundaries: guiding human development on a changing planet. Science 347. 10.1126/SCIENCE.1259855 10.1126/science.1259855 25592418

[14] Vermeulen SJ, Campbell BM, Ingram JSI (2012) Climate Change and Food systems. Annu Rev Environ Resour 37:195–222. 10.1146/ANNUREV-ENVIRON-020411-130608

[15] Aleksandrowicz L, Green R, Joy EJM et al (2016) The impacts of Dietary Change on Greenhouse Gas Emissions, Land Use, Water Use, and Health: a systematic review. PLoS ONE 11:e0165797. 10.1371/JOURNAL.PONE.0165797 27812156 10.1371/journal.pone.0165797 PMC5094759

[16] Jarmul S, Dangour AD, Green R et al (2020) Climate change mitigation through dietary change: a systematic review of empirical and modelling studies on the environmental footprints and health effects of ‘sustainable diets’. Environ Res Lett 15:123014. 10.1088/1748-9326/abc2f7 33897807 10.1088/1748-9326/abc2f7 PMC7610659

[17] Westhoek H, Rood T, van den Berg M et al (2011) The protein puzzle. The consumption and production of meat, dairy and fish in the European Union. PBL Netherlands Environmental Assessment Agency, The Hague

[18] Jafari S, Hezaveh E, Jalilpiran Y et al (2022) Plant-based diets and risk of disease mortality: a systematic review and meta-analysis of cohort studies. Crit Rev Food Sci Nutr 62:7760–7772. 10.1080/10408398.2021.1918628 33951994 10.1080/10408398.2021.1918628

[19] Payne CLR, Scarborough P, Cobiac L (2016) Do low-carbon-emission diets lead to higher nutritional quality and positive health outcomes? A systematic review of the literature. Public Health Nutr 19:2654–2661. 10.1017/S1368980016000495 26975578 10.1017/S1368980016000495 PMC10270842

[20] Willett W, Rockström J, Loken B et al (2019) Food in the Anthropocene: the EAT–Lancet Commission on healthy diets from sustainable food systems. Lancet 393:447–492. 10.1016/S0140-6736(18)31788-4 30660336 10.1016/S0140-6736(18)31788-4

[21] Neuenschwander M, Stadelmaier J, Eble J et al (2023) Substitution of animal-based with plant-based foods on cardiometabolic health and all-cause mortality: a systematic review and meta-analysis of prospective studies. BMC Med 21:404. 10.1186/s12916-023-03093-1 37968628 10.1186/s12916-023-03093-1 PMC10652524

[22] Biesbroek S, Bueno-de-Mesquita HB, Peeters PHM et al (2014) Reducing our environmental footprint and improving our health: greenhouse gas emission and land use of usual diet and mortality in EPIC-NL: a prospective cohort study. Environ Health 13:27. 10.1186/1476-069X-13-27 24708803 10.1186/1476-069X-13-27 PMC4013533

[23] Pan A, Sun Q, Bernstein AM et al (2012) Red Meat Consumption and Mortality: results from 2 prospective cohort studies. Arch Intern Med 172:555–563. 10.1001/ARCHINTERNMED.2011.2287 22412075 10.1001/archinternmed.2011.2287 PMC3712342

[24] Scarborough P, Allender S, Clarke D et al (2012) Modelling the health impact of environmentally sustainable dietary scenarios in the UK. Eur J Clin Nutr 66:710–715. 10.1038/ejcn.2012.34 22491494 10.1038/ejcn.2012.34 PMC3389618

[25] Stehfest E, Bouwman L, van Vuuren DP et al (2009) Climate benefits of changing diet. Clim Change 95:83–102. 10.1007/S10584-008-9534-6

[26] Harris F, Moss C, Joy EJM et al (2020) The Water footprint of diets: A global systematic review and Meta-analysis. Adv Nutr 11:375–386. 10.1093/advances/nmz091 31756252 10.1093/advances/nmz091 PMC7442390

[27] Meier T, Christen O (2013) Environmental impacts of dietary recommendations and dietary styles: Germany as an example. Environ Sci Technol 47:877–888. 10.1021/ES302152V 23189920 10.1021/es302152v

[28] Springmann M, Wiebe K, Mason-D’Croz D et al (2018) Health and nutritional aspects of sustainable diet strategies and their association with environmental impacts: a global modelling analysis with country-level detail. Lancet Planet Health 2:E451–E461. 10.1016/S2542-5196(18)30206-7 30318102 10.1016/S2542-5196(18)30206-7 PMC6182055

[29] Vellinga RE, van de Kamp M, Toxopeus IB et al (2019) Greenhouse Gas Emissions and Blue Water Use of Dutch Diets and its Association with Health. Sustainability 11:6027. 10.3390/SU11216027

[30] Baden MY, Liu G, Satija A et al (2019) Changes in plant-based Diet Quality and Total and cause-specific mortality. Circulation 140:979–991. 10.1161/CIRCULATIONAHA.119.041014 31401846 10.1161/CIRCULATIONAHA.119.041014 PMC6746589

[31] Kim H, Caulfield LE, Garcia-Larsen V et al (2019) Plant-based diets are Associated with a lower risk of Incident Cardiovascular Disease, Cardiovascular Disease Mortality, and all-cause mortality in a General Population of Middle-aged adults. J Am Heart Assoc 8:e012865. 10.1161/JAHA.119.012865 31387433 10.1161/JAHA.119.012865 PMC6759882

[32] Satija A, Bhupathiraju SN, Rimm EB et al (2016) Plant-based dietary patterns and incidence of type 2 diabetes in US men and women: results from three prospective cohort studies. PLoS Med 13:e1002039. 10.1371/JOURNAL.PMED.1002039 27299701 10.1371/journal.pmed.1002039 PMC4907448

[33] Li H, Zeng X, Wang Y et al (2022) A prospective study of healthful and unhealthful plant-based diet and risk of overall and cause-specific mortality. Eur J Nutr 61:387–398. 10.1007/S00394-021-02660-7 34379193 10.1007/s00394-021-02660-7

[34] Kim H, Caulfield LE, Rebholz CM (2018) Healthy plant-based diets are Associated with Lower Risk of all-cause mortality in US adults. J Nutr 148:624–631. 10.1093/JN/NXY019 29659968 10.1093/jn/nxy019 PMC6669955

[35] Thompson AS, Tresserra-Rimbau A, Karavasiloglou N et al (2023) Association of Healthful Plant-based Diet Adherence with Risk of Mortality and Major Chronic diseases among adults in the UK. JAMA Netw Open 6:e234714. 10.1001/JAMANETWORKOPEN.2023.4714 36976560 10.1001/jamanetworkopen.2023.4714 PMC10051114

[36] Kim J, Kim H, Giovannucci EL (2021) Plant-based diet quality and the risk of total and disease-specific mortality: a population-based prospective study. Clin Nutr 40:5718–5725. 10.1016/J.CLNU.2021.10.013 34749131 10.1016/j.clnu.2021.10.013

[37] Monteiro CA, Cannon G, Levy R et al (2016) NOVA. The star shines bright. World Nutr 7:28–38

[38] Monteiro CA, Cannon G, Lawrence M et al (2019) Ultra-processed foods, diet quality, and health using the NOVA classification system. FAO, Rome

[39] Mozaffarian D (2016) Dietary and Policy priorities for Cardiovascular Disease, Diabetes, and obesity. Circulation 133:187–225. 10.1161/CIRCULATIONAHA.115.018585 26746178 10.1161/CIRCULATIONAHA.115.018585 PMC4814348

[40] Suksatan W, Moradi S, Naeini F et al (2021) Ultra-processed Food Consumption and adult mortality risk: a systematic review and dose-response Meta-analysis of 207,291 participants. Nutrients 14:174. 10.3390/NU14010174 35011048 10.3390/nu14010174 PMC8747520

[41] Taneri PE, Wehrli F, Roa Diaz ZM et al (2022) Association between Ultra-processed Food Intake and all-cause mortality: a systematic review and Meta-analysis. Am J Epidemiol. 10.1093/AJE/KWAC039 35231930 10.1093/aje/kwac039

[42] Chen X, Zhang Z, Yang H et al (2020) Consumption of ultra-processed foods and health outcomes: a systematic review of epidemiological studies. Nutr J 19:86. 10.1186/S12937-020-00604-1 32819372 10.1186/s12937-020-00604-1 PMC7441617

[43] Fardet A, Rock E (2020) Ultra-processed Foods and Food System sustainability: what are the links? Sustainability 12:6280. 10.3390/SU12156280

[44] Anastasiou K, Baker P, Hadjikakou M et al (2022) A conceptual framework for understanding the environmental impacts of ultra-processed foods and implications for sustainable food systems. J Clean Prod 368:133155. 10.1016/J.JCLEPRO.2022.133155

[45] Gehring J, Touvier M, Baudry J et al (2021) Consumption of Ultra-processed Foods by Pesco-Vegetarians, vegetarians, and vegans: associations with Duration and Age at Diet initiation. J Nutr 151:120–131. 10.1093/JN/NXAA196 32692345 10.1093/jn/nxaa196

[46] Steele EM, Baraldi LG, da Costa Louzada ML et al (2016) Ultra-processed foods and added sugars in the US diet: evidence from a nationally representative cross-sectional study. BMJ Open 6:e009892. 10.1136/BMJOPEN-2015-009892 10.1136/bmjopen-2015-009892 PMC4785287 26962035

[47] Rauber F, da Costa Louzada ML, Steele EM et al (2018) Ultra-processed Food Consumption and chronic non-communicable diseases-related Dietary Nutrient Profile in the UK (2008–2014). Nutrients 10:587. 10.3390/NU10050587 29747447 10.3390/nu10050587 PMC5986467

[48] Juul F, Parekh N, Martinez-Steele E et al (2022) Ultra-processed food consumption among US adults from 2001 to 2018. Am J Clin Nutr 115:211–221. 10.1093/AJCN/NQAB305 34647997 10.1093/ajcn/nqab305

[49] Salomé M, Arrazat L, Wang J et al (2021) Contrary to ultra-processed foods, the consumption of unprocessed or minimally processed foods is associated with favorable patterns of protein intake, diet quality and lower cardiometabolic risk in French adults (INCA3). Eur J Nutr 60:4055–4067. 10.1007/S00394-021-02576-2 33966096 10.1007/s00394-021-02576-2

[50] Riboli E (1992) Nutrition and cancer: background and rationale of the European prospective investigation into Cancer and Nutrition (EPIC). Ann Oncol 3:783–791. 10.1093/oxfordjournals.annonc.a058097 1286041 10.1093/oxfordjournals.annonc.a058097

[51] Beulens JWJ, Monninkhof EM, Verschuren WMM et al (2010) Cohort Profile: the EPIC-NL study. Int J Epidemiol 39:1170–1178. 10.1093/IJE/DYP217 19483199 10.1093/ije/dyp217

[52] Boker LK, van Noord PAH, van der Schouw YT et al (2001) Prospect-EPIC Utrecht: study design and characteristics of the cohort population. Eur J Epidemiol 17:1047–1053. 10.1023/A:1020009325797 12380720 10.1023/a:1020009325797

[53] Smit HA, Verschuren WMM, Bueno-de-Mesquita HB, Seidell JC (1994) Monitoring van Risicofactoren en Gezondheid in Nederland (MORGEN-project): Doelstellingen en werkwijze. RIVM, Bilthoven

[54] Verschuren WMM, Blokstra A, Picavet HSJ, Smit HA (2008) Cohort Profile: the Doetinchem Cohort Study. Int J Epidemiol 37:1236–1241. 10.1093/IJE/DYM292 18238821 10.1093/ije/dym292

[55] Ocké MC, Bueno-de-Mesquita HB, Goddijn HE et al (1997) The Dutch EPIC food frequency questionnaire. I. description of the questionnaire, and relative validity and reproducibility for food groups. Int J Epidemiol 26:S37–S48. 10.1093/IJE/26.SUPPL_1.S37 9126532 10.1093/ije/26.suppl_1.s37

[56] Ocké MC, Bueno-de-Mesquita HB, Pols MA et al (1997) The Dutch EPIC food frequency questionnaire. II. Relative validity and reproducibility for nutrients. Int J Epidemiol 26:S49–S58. 10.1093/IJE/26.SUPPL_1.S49 9126533 10.1093/ije/26.suppl_1.s49

[57] Stichting Nederlands Voedingsstoffenbestand (1996) NEVO-tabel: Nederlands Voedingsstoffenbestand 1996. Voorlichtingsbureau voor de Voeding, Den Haag

[58] Martínez-González MA, Sánchez-Tainta A, Corella D et al (2014) A provegetarian food pattern and reduction in total mortality in the Prevención Con Dieta Mediterránea (PREDIMED) study. Am J Clin Nutr 100:320S–328S. 10.3945/AJCN.113.071431 24871477 10.3945/ajcn.113.071431

[59] Cordova R, Kliemann N, Huybrechts I et al (2021) Consumption of ultra-processed foods associated with weight gain and obesity in adults: a multi-national cohort study. Clin Nutr 40:5079–5088. 10.1016/J.CLNU.2021.08.009 34455267 10.1016/j.clnu.2021.08.009

[60] Haftenberger M, Schuit AJ, Tormo MJ et al (2002) Physical activity of subjects aged 50–64 years involved in the European prospective investigation into Cancer and Nutrition (EPIC). Public Health Nutr 5:1163–1177. 10.1079/PHN2002397 12639225 10.1079/PHN2002397

[61] Wareham NJ, Jakes RW, Rennie KL et al (2003) Validity and repeatability of a simple index derived from the short physical activity questionnaire used in the European prospective investigation into Cancer and Nutrition (EPIC) study. Public Health Nutr 6:407–413. 10.1079/PHN2002439 12795830 10.1079/PHN2002439

[62] National Institute for Public Health and the Environment (RIVM) (2021) Database Milieubelasting Voedingsmiddelen. https://www.rivm.nl/voedsel-en-voeding/duurzaam-voedsel/database-milieubelasting-voedingsmiddelen. Accessed 4 Mar 2022

[63] Vellinga RE, van den Boomgaard I, Boer JMA et al (2023) Different levels of ultra-processed food and beverage consumption and associations with environmental sustainability and all-cause mortality in EPIC-NL. Am J Clin Nutr. 10.1016/j.ajcnut.2023.05.021 37207984 10.1016/j.ajcnut.2023.05.021

[64] Hoekstra AY, Chapagain A, Martinez-Aldaya M, Mekonnen M (2011) The water footprint assessment manual; setting the global standard. Earthscan, London

[65] Knol MJ, VanderWeele TJ (2012) Recommendations for presenting analyses of effect modification and interaction. Int J Epidemiol 41:514–520. 10.1093/ije/dyr218 22253321 10.1093/ije/dyr218 PMC3324457

[66] Knol MJ, van der Tweel I, Grobbee DE et al (2007) Estimating interaction on an additive scale between continuous determinants in a logistic regression model. Int J Epidemiol 36:1111–1118. 10.1093/ije/dym157 17726040 10.1093/ije/dym157

[67] Li R, Chambless L (2007) Test for Additive Interaction in Proportional hazards models. Ann Epidemiol 17:227–236. 10.1016/j.annepidem.2006.10.009 17320789 10.1016/j.annepidem.2006.10.009

[68] Dinu M, Abbate R, Gensini G et al (2017) Vegetarian, vegan diets and multiple health outcomes: a systematic review with meta-analysis of observational studies. Crit Rev Food Sci Nutr 57:3640–3649. 10.1080/10408398.2016.1138447 26853923 10.1080/10408398.2016.1138447

[69] Jabri A, Kumar A, Verghese E et al (2021) Meta-analysis of effect of vegetarian diet on ischemic heart disease and all-cause mortality. Am J Prev Cardiol 7:100182. 10.1016/J.AJPC.2021.100182 34611632 10.1016/j.ajpc.2021.100182 PMC8387295

[70] Blanco-Rojo R, Sandoval-Insausti H, López-Garcia E et al (2019) Consumption of Ultra-processed Foods and Mortality: a national prospective cohort in Spain. Mayo Clin Proc 94:2178–2188. 10.1016/J.MAYOCP.2019.03.035 31623843 10.1016/j.mayocp.2019.03.035

[71] Bonaccio M, Di Castelnuovo A, Costanzo S et al (2021) Ultra-processed food consumption is associated with increased risk of all-cause and cardiovascular mortality in the Moli-Sani Study. Am J Clin Nutr 113:446–455. 10.1093/AJCN/NQAA299 33333551 10.1093/ajcn/nqaa299

[72] Kim H, Hu EA, Rebholz CM (2019) Ultra-processed food intake and mortality in the USA: results from the Third National Health and Nutrition Examination Survey (NHANES III, 1988–1994). Public Health Nutr 22:1777–1785. 10.1017/S1368980018003890 30789115 10.1017/S1368980018003890 PMC6554067

[73] Rico-Campà A, Martínez-González M, Alvarez-Alvarez I et al (2019) Association between consumption of ultra-processed foods and all cause mortality: SUN prospective cohort study. BMJ 365. 10.1136/bmj.l1949 10.1136/bmj.l1949 PMC6538973 31142450

[74] Schnabel L, Kesse-Guyot E, Allès B et al (2019) Association between Ultraprocessed Food Consumption and Risk of Mortality among Middle-aged adults in France. JAMA Intern Med 179:490–498. 10.1001/JAMAINTERNMED.2018.7289 30742202 10.1001/jamainternmed.2018.7289 PMC6450295

[75] Romero Ferreiro C, Martín-Arriscado Arroba C, Cancelas Navia P et al (2021) Ultra-processed food intake and all-cause mortality: DRECE cohort study. Public Health Nutr 25:1–10. 10.1017/S1368980021003256 34348832 10.1017/S1368980021003256 PMC9991788

[76] Pinho MGM, Lakerveld J, Harbers MC et al (2021) Ultra-processed food consumption patterns among older adults in the Netherlands and the role of the food environment. Eur J Nutr 60:2567–2580. 10.1007/s00394-020-02436-5 33236180 10.1007/s00394-020-02436-5 PMC8275501

[77] Vellinga RE, van Bakel M, Biesbroek S et al (2022) Evaluation of foods, drinks and diets in the Netherlands according to the degree of processing for nutritional quality, environmental impact and food costs. BMC Public Health 22:877. 10.1186/S12889-022-13282-X 35501799 10.1186/s12889-022-13282-x PMC9063197

[78] Marino M, Puppo F, Del Bo’ C et al (2021) A systematic review of worldwide consumption of ultra-processed foods: findings and criticisms. Nutrients 13:2778. 10.3390/NU13082778 34444936 10.3390/nu13082778 PMC8398521

[79] Seferidi P, Scrinis G, Huybrechts I et al (2020) The neglected environmental impacts of ultra-processed foods. Lancet Planet Health 4:e437–e438. 10.1016/S2542-5196(20)30177-7 33038314 10.1016/S2542-5196(20)30177-7

[80] MacDiarmid JI (2022) The food system and climate change: are plant-based diets becoming unhealthy and less environmentally sustainable? Proc Nutr Soc 81:162–167. 10.1017/S0029665121003712 35156593 10.1017/S0029665121003712

[81] Kesse-Guyot E, Pointereau P, Brunin J et al (2023) Trade-offs between blue water use and greenhouse gas emissions related to food systems: an optimization study for French adults. Sustain Prod Consum 42:33–43. 10.1016/j.spc.2023.09.008

[82] Ringler C, Agbonlahor M, Baye K et al (2023) Water for Food Systems and Nutrition. Science and innovations for Food systems Transformation. Springer, Cham, pp 497–509

[83] Freedman LS, Schatzkin A, Midthune D, Kipnis V (2011) Dealing with dietary measurement error in nutritional cohort studies. J Natl Cancer Inst 103:1086–1092. 10.1093/jnci/djr189 21653922 10.1093/jnci/djr189 PMC3143422

[84] Gibney MJ, Forde CG, Mullally D, Gibney ER (2017) Ultra-processed foods in human health: a critical appraisal. Am J Clin Nutr 106:717–724. 10.3945/AJCN.117.160440 28793996 10.3945/ajcn.117.160440

[85] Duan MJ, Vinke PC, Navis G et al (2022) Ultra-processed food and incident type 2 diabetes: studying the underlying consumption patterns to unravel the health effects of this heterogeneous food category in the prospective Lifelines cohort. BMC Med 20:7. 10.1186/S12916-021-02200-4 35022060 10.1186/s12916-021-02200-4 PMC8756643

[86] Kelly B, Jacoby E (2018) Public Health Nutrition special issue on ultra-processed foods. Public Health Nutr 21:1–4. 10.1017/S1368980017002853 29227217 10.1017/S1368980017002853 PMC10260826

[87] Moubarac JC, Parra DC, Cannon G, Monteiro CA (2014) Food classification systems based on Food Processing: significance and implications for policies and actions: a systematic Literature Review and Assessment. Curr Obes Rep 3:256–272. 10.1007/S13679-014-0092-0 26626606 10.1007/s13679-014-0092-0

