# Supplementary material for: The role of ultra-processed foods in plant-based diets: associations with human health and environmental sustainability
Source: Eur J Nutr. 2024 Aug 24;63(8):2957–73. doi: 10.1007/s00394-024-03477-w (PMC11519232; doi:10.1007/s00394-024-03477-w)
Supplement: Supplementary file 1 — Supplementary Material 1 [file 394_2024_3477_MOESM1_ESM.pdf]

Supplementary information

European Journal of Nutrition

The Role of Ultra-processed Foods in Plant-based Diets: Associations with Human Health and Environmental Sustainability

Merel C. Daas[1], Reina E. Vellinga[1,2], Maria Gabriela M. Pinho[3], Jolanda M.A. Boer[2], W.M. Monique Verschuren[2,4], Yvonne T. van der Schouw[4], Pieter van 't Veer[1], Sander Biesbroek[1]

[1]Division of Human Nutrition and Health, Wageningen University & Research, P.O. Box 17, 6700 AA Wageningen, The Netherlands

[2]Centre for Prevention, Lifestyle and Health, National Institute for Public Health and the Environment (RIVM), Antonie van Leeuwenhoeklaan 9, 3721 MA Bilthoven, The Netherlands

[3]Copernicus Institute of Sustainable Development, Utrecht University, Princetonlaan 8a, 3584 CB Utrecht, The Netherlands

[4]Julius Center for Health Sciences and Primary Care, University Medical Center Utrecht, Universiteitsweg 100, 3584 CG Utrecht, The Netherlands

Address correspondence to Merel C. Daas (e-mail: merel.daas@wur.nl)

Supplemental Table 1 Food groups with included foods and scoring criteria for the hPDI and uPDI.

Food groups[a]	Included foods	hPDI[b]	uPDI[b]
**Healthy plant-based foods**			
1. Vegetables	Mushrooms, cucumber, leek, onion, chicory, carrots, garlic, beetroot, tomatoes, tomato sauce, tomato paste, lettuce, cabbage, bell peppers, green beans, peas, spinach, endive, pickles	Positive[c]	Reverse[d]
2. Fruit	Strawberries, bananas, grapes, cherries, peaches, apple sauce, kiwi, melons, apples, pears, citrus fruits, olives	Positive	Reverse
3. Legumes	Legumes, tempeh, tofu		
4. Whole grains	Brown rice, breakfast cereals, wholegrain bread, brown bread, rye bread, grains (millet, buckwheat, groats)	Positive	Reverse
5. Nuts and seeds	Peanut sauce, peanut butter, nuts, peanuts	Positive	Reverse
6. Vegetable oils and fats	Peanut oil, corn (germ) oil, soybean oil, margarine, sunflower oil, olive oil, safflower oil, palm oil, oil blend, liquid fry and roast, melting gravy, frying fat (plant-based)	Positive	Reverse
7. Tea and coffee	Tea, coffee	Positive	Reverse
**Unhealthy plant-based foods**			
8. Refined grains	White rice, croissants, rusk, crispbread, toast, pasta, gingerbread, pretzels, dough	Reverse	Positive
9. Potatoes	French fries, boiled potatoes, baked potatoes	Reverse	Positive
10. Juices	Apple juice, orange juice, grapefruit juice, other fruit juice, vegetable juice	Reverse	Positive
11. (Sugar) sweetened beverages	Coke, (diet) sodas, lemonade	Reverse	Positive
12. Sweets and desserts	Sugar, honey, sirop, sweet spread, chocolate, chocolate sprinkles, liquorice, candy, pie, cakes, cookies	Reverse	Positive
**Animal-based foods**			
13. Meat	Beef, pork, chicken, (smoked) sausages, minced meat, cooked ham, roasted beef, smoked meat, casserole rib, liver, bacon, cervelat, organ meats, processed meat	Reverse	Reverse
14. Animal fats	Lard, (herb) butter, frying fat (animal-based)	Reverse	Reverse
15. Eggs	Eggs	Reverse	Reverse
16. Fish and seafood	Fish, shellfish, sardines, shrimps, fish fingers	Reverse	Reverse
17. Dairy products	Milk, coffee creamer (powder), (drinking) yogurt, whipped cream, creamy salad dressing, cheese, cream cheese, cheese spread, chocolate milk, custard, pudding, cottage cheese, ice cream	Reverse	Reverse
18. Miscellaneous animal-based foods	Spring rolls, Russian salad, mayonnaise, pizza, vegetarian schnitzel with eggs, hot sauces, soups	Reverse	Reverse

hPDI healthful plant-based diet index, *uPDI* unhealthful plant-based diet index.

[a]Each food item of the FFQ was assigned to a food group. The consumption (g/day) of each food group per individual was transformed into sex-specific quintiles and given a score between 1 and 5.

[b]The hPDI and uPDI were calculated by summing the scores of all food groups with a potential range of 18-90.

[c]For positive scores, the highest scores were attributed to individuals in the highest consumption quintiles (5 points for the 5th quintile to 1 point for the 1st quintile).

[d]For reverse scores, the highest scores were attributed to individuals in the lowest consumption quintiles (5 points for the 1st quintile to 1 point for the 5th quintile).

Supplemental Table 2 Sensitivity analysis in which alcoholic beverages were excluded from the NOVA classification. Associations between the PDI indices (per 10-point increase) and all-cause mortality risk, GHGE, and BWC by UPF consumption in the EPIC-NL cohort.

	**UPF**								**Measure of additive interaction**		
	Q1 (≤ 226 g/2000 kcal)		Q2 (226-299 g/2000 kcal)		Q3 (299-404 g/2000 kcal)		Q4 (> 404 g/2000 kcal)				
	HR	95% CI	HR	95% CI	HR	95% CI	HR	95% CI	RERI[a,b]	95% CI	
**All-cause mortality**											
hPDI[b]	0.95	0.86, 1.06	0.81	0.73, 0.91	0.88	0.77, 1.00	0.93	0.82, 1.07	0.017	-0.031, 0.065	
uPDI[b]	1.08	0.98, 1.20	1.09	0.98, 1.22	1.01	0.90, 1.14	1.02	0.90, 1.16	0.005	-0.020, 0.030	
	ΔMean	95% CI	ΔMean	95% CI	ΔMean	95% CI	ΔMean	95% CI	β_3[c,d]	95% CI	*P* value
**GHGE (kg CO_2-eq/day)**											
hPDI[d]	-0.38	-0.42, -0.35	-0.39	-0.42, -0.36	-0.36	-0.40, -0.33	-0.29	-0.32, -0.25	-0.0001	-0.0002, 0.0000	0.162
uPDI[d]	-0.50	-0.54, -0.47	-0.51	-0.54, -0.48	-0.53	-0.56, -0.50	-0.60	-0.63, -0.57	-0.0002	-0.0003, -0.0001	< 0.001
**BWC (m^3/day)**											
hPDI[d]	11.1	9.3, 12.9	7.6	5.9, 9.3	7.6	6.0, 9.3	8.2	6.5, 10.0	-0.006	-0.011, -0.002	0.010
uPDI[d]	-16.1	-17.9, -14.3	-12.7	-14.3, -11.0	-12.8	-14.4, -11.2	-14.2	-15.9, -12.6	0.006	0.002, 0.010	0.006

BWC blue water consumption, *CI* confidence interval, *GHGE* greenhouse gas emissions, *HR* hazard ratio, *hPDI* healthful plant-based diet index, *Q* quartile, *uPDI* unhealthful plant-based diet index, *UPF* ultra-processed foods.

[a]Relative excess all-cause mortality risk due to interaction between a 10-point decrease in the hPDI score or 10-point increase in the uPDI score and a 100 g/2000 kcal increase in UPF consumption. The hPDI was recoded into a risk factor for correct calculation of the RERI.

[b]Adjusted for age, sex, total energy intake, educational level, smoking status, physical activity level, and alcohol consumption.

[c]Absolute excess GHGE or BWC due to interaction between a 10-point increase in the hPDI or uPDI score and a 100 g/2000 kcal decrease in UPF consumption.

[d]Adjusted for age, sex, and total energy intake.

Supplemental Table 3 Consumption of food groups stratified for NOVA classes and their contribution to environmental impacts according to quartiles of the uPDI in the EPIC-NL cohort.

	uPDI											
	Q1 (≤ 49 points)			Q2 (49-54 points)			Q3 (54-58 points)			Q4 (> 58 points)		
**Food groups**	Weight[a]	%GHGE	%BWC	Weight[a]	%GHGE	%BWC	Weight[a]	%GHGE	%BWC	Weight[a]	%GHGE	%BWC
**Healthy plant-based foods**												
Vegetables	158 [119 - 208]	4.1%	5.8%	140 [105 - 184]	3.9%	5.4%	125 [94 - 165]	3.6%	5.1%	106 [78 - 141]	3.2%	4.7%
MPF	157 [118 - 207]	4.0%	5.6%	139 [105 - 184]	3.8%	5.3%	124 [94 - 164]	3.5%	5.0%	106 [78 - 141]	3.2%	4.6%
PCI	0.0 [0.0 - 0.7]	0.1%	0.2%	0.0 [0.0 - 0.6]	0.1%	0.2%	0.0 [0.0 - 0.2]	0.1%	0.1%	0.0 [0.0 - 0.0]	0.0%	0.1%
Fruit	233 [137 - 343]	5.6%	30.5%	188 [108 - 303]	5.2%	27.6%	153 [86 - 260]	4.6%	24.7%	113 [58 - 195]	3.9%	20.3%
MPF	233 [137 - 343]	5.6%	30.5%	188 [108 - 303]	5.2%	27.6%	153 [86 - 260]	4.6%	24.7%	113 [58 - 195]	3.9%	20.3%
Legumes	11 [5 - 18]	0.5%	0.6%	8.0 [3.6 - 14.8]	0.4%	0.5%	6.2 [2.5 - 12.3]	0.4%	0.4%	4.4 [1.3 - 9.4]	0.3%	0.4%
MPF	11 [5 - 18]	0.5%	0.6%	8.0 [3.6 - 14.8]	0.4%	0.5%	6.2 [2.5 - 12.3]	0.4%	0.4%	4.4 [1.3 - 9.4]	0.3%	0.4%
Whole grains	132 [103 - 161]	2.5%	2.1%	123 [91 - 155]	2.4%	2.0%	109 [72 - 145]	2.2%	1.9%	83 [35 - 123]	1.8%	1.6%
MPF	3.5 [0.0 - 11.8]	0.2%	0.8%	2.9 [0.0 - 10.1]	0.2%	0.7%	2.3 [0.0 - 8.6]	0.2%	0.7%	1.8 [0.1 - 6.5]	0.2%	0.6%
PCI	112 [83 - 141]	1.9%	1.1%	105 [73 - 137]	1.9%	1.0%	92 [55 - 128]	1.7%	1.0%	68 [15 - 108]	1.4%	0.8%
UPF	2.4 [0.0 - 14.3]	0.3%	0.3%	1.5 [0.0 - 11.2]	0.3%	0.2%	1.0 [0.0 - 9.3]	0.3%	0.2%	0.4 [0.0 - 6.5]	0.2%	0.2%
Nuts and seeds	7.1 [2.9 - 13.9]	1.2%	1.2%	6.2 [2.4 - 12.6]	1.2%	1.2%	5.7 [2.2 - 11.5]	1.1%	1.2%	5.1 [2.0 - 10.6]	1.1%	1.2%
MPF	0.0 [0.0 - 0.0]	0.0%	0.3%	0.0 [0.0 - 0.0]	0.0%	0.3%	0.0 [0.0 - 0.0]	0.0%	0.3%	0.0 [0.0 - 0.1]	0.0%	0.3%
PF	5.5 [1.8 - 12.3]	1.1%	0.9%	4.6 [1.5 - 10.6]	1.1%	0.8%	4.0 [1.2 - 9.3]	1.0%	0.8%	3.5 [1.0 - 8.3]	1.0%	0.8%
UPF	0.3 [0.0 - 1.4]	0.0%	0.0%	0.3 [0.0 - 1.5]	0.1%	0.0%	0.4 [0.0 - 1.6]	0.1%	0.1%	0.4 [0.0 - 1.7]	0.1%	0.1%
Vegetable oils and fats	21 [14 - 28]	2.0%	3.0%	19 [12 - 27]	1.9%	2.7%	18 [11 - 26]	1.8%	2.6%	15 [9 - 23]	1.7%	2.4%
PCI	4.6 [2.0 - 7.8]	0.9%	2.3%	3.7 [1.5 - 6.8]	0.8%	2.1%	3.1 [1.1 - 5.9]	0.7%	1.9%	2.2 [0.6 - 4.7]	0.6%	1.6%
UPF	16 [8 - 23]	1.1%	0.7%	14 [7 - 22]	1.1%	0.7%	14 [7 - 212]	1.1%	0.7%	12 [6 - 20]	1.1%	0.7%
Tea and coffee	955 [714 - 1244]	4.1%	13.0%	876 [646 - 1163]	4.0%	12.1%	794 [565 - 1071]	3.8%	11.3%	662 [442 - 923]	3.5%	10.3%
MPF	955 [714 - 1244]	4.1%	13.0%	876 [646 - 1163]	4.0%	12.1%	794 [565 - 1071]	3.8%	11.3%	662 [442 - 923]	3.5%	10.3%
**Unhealthy plant-based foods**												
Refined grains	53 [35 - 76]	1.7%	1.9%	65 [41 - 93]	2.2%	2.5%	77 [50 - 113]	2.7%	3.2%	100 [65 - 146]	3.6%	4.2%
MPF	24 [12 - 41]	0.8%	1.2%	28 [13 - 49]	1.0%	1.6%	32 [15 - 56]	1.2%	2.1%	35 [17 - 60]	1.5%	2.6%
PCI	0.0 [0.0 - 1.6]	0.1%	0.1%	0.0 [0.0 - 5.6]	0.2%	0.1%	0.0 [0.0 - 17.6]	0.4%	0.2%	7.3 [0.0 - 58.2]	0.8%	0.5%
UPF	20 [11 - 33]	0.8%	0.7%	23 [12 - 36]	1.0%	0.8%	23 [11 - 39]	1.1%	0.9%	24 [12 - 41]	1.4%	1.1%
Potatoes	79 [52 - 117]	2.0%	1.0%	87 [57 - 128]	2.6%	1.3%	95 [62 - 140]	3.2%	1.7%	106 [70 - 148]	4.5%	2.5%
MPF	71 [45 - 109]	1.3%	0.7%	76 [47 - 115]	1.4%	0.7%	79 [48 - 122]	1.5%	0.8%	79 [47 - 120]	1.6%	0.9%
PF	0.0 [0.0 - 10.6]	0.7%	0.4%	0.5 [0.0 - 19.0]	1.2%	0.6%	8.0 [0.0 - 25.7]	1.7%	0.9%	20 [0 - 37]	2.9%	1.6%
Juices	31 [5 - 97]	1.2%	10.7%	48 [12 - 127]	1.6%	14.4%	54 [13 - 135]	1.8%	16.6%	65 [18 - 146]	2.2%	20.4%
MPF	26 [4 - 81]	1.1%	10.1%	40 [9 - 109]	1.5%	13.6%	45 [11 - 116]	1.7%	15.6%	54 [15 - 127]	2.0%	19.1%
UPF	3.1 [0.5 - 9.6]	0.1%	0.6%	5.0 [1.1 - 11.9]	0.1%	0.8%	5.5 [1.2 - 13.3]	0.1%	1.0%	6.4 [1.7 - 15.5]	0.2%	1.2%
(Sugar) sweetened beverages	31 [4 - 83]	0.6%	0.3%	57 [13 - 135]	0.9%	0.4%	83 [22 - 175]	1.3%	0.6%	141 [57 - 261]	2.1%	1.0%
UPF	31 [4 - 83]	0.6%	0.3%	57 [13 - 135]	0.9%	0.4%	83 [22 - 175]	1.3%	0.6%	141 [57 - 261]	2.1%	1.0%
Sweets and desserts	42 [27 - 56]	2.0%	2.5%	51 [33 - 71]	2.5%	3.1%	58 [38 - 81]	2.9%	3.6%	69 [47 - 93]	3.8%	4.7%
PCI	0.7 [0.0 - 7.5]	0.1%	0.1%	1.4 [0.0 - 13.5]	0.1%	0.1%	3.5 [0.1 - 20.2]	0.2%	0.1%	9.3 [0.3 - 27.8]	0.3%	0.2%
PF	0.0 [0.0 - 6.2]	0.1%	0.3%	0.0 [0.0 - 5.1]	0.1%	0.3%	0.0 [0.0 - 2.1]	0.1%	0.3%	0.0 [0.0 - 0.0]	0.1%	0.2%
UPF	32 [19 - 46]	1.8%	2.1%	37 [22 - 54]	2.2%	2.7%	41 [24 - 61]	2.6%	3.2%	48 [28 - 71]	3.4%	4.3%

**Animal-based foods**												
Meat	110 [76 - 142]	33.6%	10.4%	104 [70 - 136]	33.4%	10.4%	102 [70 - 134]	34.0%	10.9%	99 [68 - 131]	34.3%	11.4%
MPF	76 [50 - 102]	26.3%	8.2%	72 [47 - 98]	26.4%	8.2%	70 [46 - 96]	26.9%	8.6%	67 [43 - 92]	26.9%	8.9%
UPF	28 [15 - 44]	7.3%	2.2%	26 [14 - 42]	7.0%	2.2%	26 [14 - 43]	7.1%	2.3%	27 [15 - 43]	7.4%	2.5%
Animal fats	3.3 [2.0 - 6.9]	1.1%	0.3%	2.9 [1.7 - 5.6]	1.1%	0.3%	2.6 [1.5 - 4.9]	1.0%	0.3%	2.1 [1.2 - 3.7]	0.9%	0.3%
PCI	3.3 [2.0 - 6.5]	1.1%	0.3%	2.8 [1.7 - 5.3]	1.0%	0.3%	2.6 [1.5 - 4.7]	1.0%	0.3%	2.1 [1.2 - 3.6]	0.9%	0.3%
Eggs	17 [11 - 25]	1.4%	1.2%	14 [8 - 22]	1.3%	1.1%	12 [7 - 20]	1.2%	1.0%	9.9 [5.1 - 16.7]	1.1%	1.0%
MPF	17 [11 - 25]	1.4%	1.2%	14 [8 - 22]	1.3%	1.1%	12 [7 - 20]	1.2%	1.0%	9.9 [5.1 - 16.7]	1.1%	1.0%
Fish and seafood	11 [5 - 18]	2.1%	0.4%	8.4 [3.5 - 15.6]	1.9%	0.4%	6.2 [2.5 - 12.9]	1.7%	0.3%	4.1 [1.4 - 9.3]	1.4%	0.3%
MPF	8.0 [3.7 - 12.6]	1.7%	0.3%	6.3 [2.5 - 11.6]	1.6%	0.3%	5.0 [2.0 - 10.5]	1.5%	0.3%	3.6 [1.2 - 8.2]	1.3%	0.2%
PF	0.0 [0.0 - 0.1]	0.0%	0.0%	0.0 [0.0 - 0.1]	0.0%	0.0%	0.0 [0.0 - 0.0]	0.0%	0.0%	0.0 [0.0 - 0.0]	0.0%	0.0%
UPF	0.9 [0.0 - 5.8]	0.4%	0.1%	0.0 [0.0 - 3.2]	0.3%	0.1%	0.0 [0.0 - 1.1]	0.2%	0.0%	0.0 [0.0 - 0.0]	0.1%	0.0%
Dairy products	474 [315 - 655]	23.8%	6.7%	419 [253 - 603]	23.4%	6.6%	371 [216 - 552]	22.6%	6.5%	295 [166 - 478]	21.2%	6.3%
MPF	374 [227 - 547]	13.4%	3.7%	316 [169 - 490]	12.7%	3.5%	273 [133 - 439]	12.0%	3.4%	204 [91 - 366]	10.7%	3.2%
PCI	1.8 [0.9 - 3.4]	0.2%	0.1%	1.8 [0.8 - 3.4]	0.2%	0.1%	1.7 [0.8 - 3.3]	0.2%	0.1%	1.7 [0.7 - 3.1]	0.2%	0.1%
PF	35 [22 - 51]	7.9%	2.2%	32 [20 - 49]	8.0%	2.2%	30 [17 - 47]	7.7%	2.2%	26 [12 - 43]	7.5%	2.2%
UPF	47 [25 - 74]	2.3%	0.7%	48 [24 - 77]	2.5%	0.7%	48 [24 - 79]	2.7%	0.8%	47 [22 - 80]	2.9%	0.9%
Miscellaneous[b]	88 [51 - 141]	7.1%	4.1%	70 [41 - 117]	6.4%	3.6%	64 [38 - 109]	6.3%	3.6%	58 [35 - 96]	6.0%	3.5%
MPF	70 [34 - 121]	6.1%	3.6%	48 [25 - 94]	5.1%	3.0%	41 [20 - 83]	4.8%	2.9%	32 [16 - 65]	4.1%	2.6%
PCI	12 [6 - 23]	1.1%	0.5%	13 [6 - 26]	1.3%	0.6%	15 [7 - 29]	1.5%	0.7%	19 [8 - 34]	1.9%	0.9%
**Other foods**												
Alcoholic beverages	75 [15 - 188]	3.4%	3.9%	66 [11 - 187]	3.5%	3.9%	61 [9 - 197]	3.5%	3.9%	41 [4 - 171]	3.1%	3.2%
PF	39 [5 - 133]	2.4%	3.0%	35 [4 - 133]	2.5%	3.0%	33 [3 - 147]	2.6%	3.0%	24 [1 - 128]	2.4%	2.5%
UPF	2.8 [0.0 - 21.0]	1.0%	1.0%	2.0 [0.0 - 16.1]	1.0%	0.9%	1.1 [0.0 - 11.7]	0.9%	0.8%	0.3 [0.0 - 6.1]	0.7%	0.6%
Drinking water	330 [150 - 612]	0.3%	0.4%	314 [140 - 592]	0.4%	0.5%	282 [126 - 533]	0.5%	0.5%	237 [99 - 469]	0.5%	0.5%
MPF	330 [150 - 612]	0.3%	0.4%	314 [140 - 592]	0.4%	0.5%	282 [126 - 533]	0.5%	0.5%	237 [99 - 469]	0.5%	0.5%

BWC blue water consumption, *GHGE* greenhouse gas emissions, *MPF* unprocessed/minimally processed foods, *PCI* processed culinary ingredients, *PF* processed foods, *Q* quartile, *uPDI* unhealthful plant-based diet index, *UPF* ultra-processed foods.

[a]Variables are adjusted for total energy intake, expressed per 2000 kcal, and presented as median with interquartile range [25 - 75th percentile].

[b]Mixed dishes that contain substantial amounts of animal sourced ingredients (e.g. soups, pizza, and mayonnaise). Supplemental Table 1 displays detailed information on included food items.

Supplemental Table 4 Consumption of food groups stratified for NOVA classes and their contribution to environmental impacts according to quartiles of the hPDI in the EPIC-NL cohort.

	**hPDI**											
	Q1 (≤ 50 points)			Q2 (50-54 points)			Q3 (54-58 points)			Q4 (> 58 points)		
**Food groups**	Weight[a]	%GHGE	%BWC	Weight[a]	%GHGE	%BWC	Weight[a]	%GHGE	%BWC	Weight[a]	%GHGE	%BWC
**Healthy plant-based foods**												
Vegetables	118 [87 - 157]	3.2%	4.9%	133 [99 - 177]	3.7%	5.3%	139 [103 - 186]	3.9%	5.5%	143 [105 - 190]	4.3%	5.7%
MPF	117 [87 - 157]	3.1%	4.7%	133 [99 - 177]	3.6%	5.1%	139 [102 - 185]	3.9%	5.3%	143 [105 - 190]	4.2%	5.6%
PCI	0.0 [0.0 - 0.1]	0.1%	0.1%	0.0 [0.0 - 0.4]	0.1%	0.1%	0.0 [0.0 - 0.5]	0.1%	0.2%	0.0 [0.0 - 0.5]	0.1%	0.2%
Fruit	124 [66 - 209]	3.6%	20.4%	167 [95 - 281]	4.7%	25.7%	194 [108 - 312]	5.4%	28.3%	222 [124 - 336]	6.2%	31.4%
MPF	124 [66 - 209]	3.6%	20.4%	167 [95 - 281]	4.7%	25.7%	194 [108 - 312]	5.4%	28.3%	222 [124 - 336]	6.2%	31.4%
Legumes	4.8 [1.6 - 10.1]	0.3%	0.4%	6.7 [2.7 - 12.9]	0.3%	0.4%	8.3 [3.6 - 15.2]	0.4%	0.5%	10 [5 - 18]	0.6%	0.6%
MPF	4.8 [1.6 - 10.1]	0.3%	0.4%	6.7 [2.7 - 12.9]	0.3%	0.4%	8.3 [3.6 - 15.2]	0.4%	0.5%	10 [5 - 18]	0.6%	0.6%
Whole grains	82 [39 - 116]	1.5%	1.5%	112 [78 - 143]	2.1%	1.9%	125 [95 - 157]	2.5%	2.1%	143 [112 - 174]	3.0%	2.4%
MPF	1.9 [0.0 - 7.1]	0.2%	0.6%	2.3 [0.0 - 9.0]	0.2%	0.7%	3.1 [0.1 - 10.5]	0.2%	0.8%	3.3 [0.2 - 11.7]	0.3%	0.8%
PCI	67 [21 - 101]	1.2%	0.7%	95 [62 - 127]	1.7%	1.0%	107 [75 - 138]	1.9%	1.1%	122 [91 - 153]	2.4%	1.2%
UPF	0.5 [0.0 - 6.9]	0.2%	0.2%	1.0 [0.0 - 9.4]	0.3%	0.2%	1.6 [0.0 - 12.5]	0.3%	0.3%	2.4 [0.0 - 14.5]	0.4%	0.3%
Nuts and seeds	4.4 [1.7 - 8.6]	0.8%	0.8%	5.5 [2.1 - 10.9]	1.0%	1.0%	6.7 [2.6 - 13.1]	1.2%	1.2%	9.2 [3.9 - 17.8]	1.8%	1.8%
MPF	0.0 [0.0 - 0.0]	0.0%	0.2%	0.0 [0.0 - 0.0]	0.0%	0.2%	0.0 [0.0 - 0.1]	0.0%	0.3%	0.0 [0.0 - 0.2]	0.1%	0.5%
PF	2.9 [0.9 - 6.7]	0.7%	0.6%	4.0 [1.2 - 8.9]	0.9%	0.7%	4.9 [1.7 - 11.1]	1.1%	0.9%	7.4 [2.5 - 15.8]	1.7%	1.2%
UPF	0.3 [0.0 - 1.6]	0.1%	0.1%	0.3 [0.0 - 1.5]	0.1%	0.0%	0.3 [0.0 - 1.5]	0.1%	0.0%	0.4 [0.0 - 1.4]	0.1%	0.0%
Vegetable oils and fats	16 [10 - 23]	1.5%	2.6%	18 [12 - 26]	1.8%	2.6%	19 [13 - 27]	1.9%	2.7%	22 [14 - 29]	2.2%	3.0%
PCI	2.9 [1.0 - 5.7]	0.6%	1.9%	3.3 [1.2 - 6.3]	0.7%	1.9%	3.6 [1.4 - 6.6]	0.8%	2.0%	3.8 [1.5 - 7.1]	0.9%	2.2%
UPF	11 [5 - 19]	0.9%	0.6%	14 [7 - 21]	1.1%	0.7%	15 [7 - 23]	1.1%	0.7%	17 [9 - 24]	1.3%	0.8%
Tea and coffee	705 [481 - 979]	3.3%	10.1%	847 [604 - 1133]	3.9%	11.7%	884 [644 - 1173]	4.1%	12.4%	895 [668 - 1194]	4.4%	13.4%
MPF	705 [481 - 979]	3.3%	10.1%	847 [604 - 1133]	3.9%	11.7%	884 [644 - 1173]	4.1%	12.4%	895 [668 - 1194]	4.4%	13.4%
**Unhealthy plant-based foods**												
Refined grains	90 [58 - 134]	2.9%	3.5%	71 [45 - 106]	2.5%	2.8%	64 [41 - 95]	2.3%	2.6%	57 [37 - 82]	2.2%	2.3%
MPF	32 [15 - 57]	1.2%	2.2%	29 [14 - 52]	1.1%	1.8%	28 [13 - 49]	1.1%	1.7%	27 [13 - 47]	1.1%	1.5%
PCI	4.6 [0.0 - 48.1]	0.6%	0.4%	0.0 [0.0 - 11.4]	0.3%	0.2%	0.0 [0.0 - 5.3]	0.2%	0.1%	0.0 [0.0 - 2.0]	0.1%	0.1%
UPF	24 [12 - 39]	1.1%	0.9%	24 [12 - 38]	1.1%	0.8%	23 [11 - 36]	1.0%	0.8%	21 [11 - 34]	1.0%	0.7%
Potatoes	103 [69 - 146]	3.6%	2.1%	94 [61 - 134]	2.9%	1.6%	88 [56 - 129]	2.7%	1.4%	78 [50 - 119]	2.5%	1.2%
MPF	81 [50 - 122]	1.4%	0.9%	79 [49 - 117]	1.4%	0.8%	75 [46 - 116]	1.4%	0.8%	69 [42 - 106]	1.4%	0.7%
PF	13 [0 - 33]	2.2%	1.2%	3.9 [0.0 - 23.7]	1.5%	0.8%	1.3 [0.0 - 19.9]	1.3%	0.6%	0.8 [0.0 - 16.5]	1.1%	0.5%
Juices	72 [23 - 150]	2.0%	19.4%	53 [13 - 133]	1.7%	15.7%	44 [9 - 120]	1.6%	13.9%	27 [4 - 88]	1.3%	10.7%
MPF	58 [19 - 131]	1.8%	18.3%	44 [11 - 114]	1.6%	14.8%	36 [7 - 101]	1.5%	13.1%	23 [3 - 73]	1.2%	10.1%
UPF	7.2 [2.1 - 15.8]	0.1%	1.2%	5.5 [1.2 - 13.1]	0.1%	0.9%	4.3 [0.8 - 11.3]	0.1%	0.8%	2.8 [0.4 - 8.7]	0.1%	0.6%
(Sugar) sweetened beverages	125 [49 - 238]	1.7%	0.8%	77 [20 - 168]	1.2%	0.6%	53 [12 - 132]	0.9%	0.4%	31 [4 - 84]	0.7%	0.3%
UPF	125 [49 - 238]	1.7%	0.8%	77 [20 - 168]	1.2%	0.6%	53 [12 - 132]	0.9%	0.4%	31 [4 - 84]	0.7%	0.3%
Sweets and desserts	56 [36 - 78]	2.7%	3.5%	54 [34 - 77]	2.7%	3.4%	53 [34 - 76]	2.7%	3.3%	51 [33 - 73]	2.8%	3.3%
PCI	5.2 [0.1 - 22.5]	0.2%	0.1%	1.8 [0.0 - 16.8]	0.2%	0.1%	1.7 [0.0 - 14.4]	0.2%	0.1%	1.5 [0.0 - 13.2]	0.1%	0.1%
PF	0.0 [0.0 - 0.3]	0.1%	0.2%	0.0 [0.0 - 3.5]	0.1%	0.3%	0.0 [0.0 - 4.7]	0.1%	0.3%	0.0 [0.0 - 6.3]	0.2%	0.3%
UPF	38 [22 - 58]	2.4%	3.2%	39 [23 - 58]	2.4%	3.0%	38 [23 - 58]	2.4%	2.9%	37 [23 - 56]	2.5%	2.9%

**Animal-based foods**												
Meat	119 [88 - 149]	36.6%	12.7%	107 [75 - 138]	34.3%	11.0%	99 [67 - 131]	32.9%	10.1%	86 [54 - 117]	30.1%	8.8%
MPF	81 [57 - 106]	28.8%	10.0%	74 [49 - 100]	27.0%	8.7%	69 [44 - 95]	26.1%	8.0%	59 [35 - 83]	23.6%	6.9%
UPF	32 [19 - 49]	7.8%	2.7%	28 [16 - 44]	7.3%	2.3%	25 [13 - 40]	6.9%	2.1%	22 [10 - 37]	6.5%	1.9%
Animal fats	3.1 [1.6 - 7.3]	1.2%	0.4%	2.7 [1.6 - 5.5]	1.0%	0.3%	2.6 [1.5 - 4.6]	0.9%	0.3%	2.6 [1.6 - 4.1]	0.9%	0.2%
PCI	3.0 [1.6 - 6.9]	1.2%	0.4%	2.7 [1.6 - 5.2]	1.0%	0.3%	2.6 [1.5 - 4.4]	0.9%	0.3%	2.6 [1.6 - 4.0]	0.9%	0.2%
Eggs	17 [11 - 26]	1.5%	1.4%	15 [8 - 22]	1.3%	1.1%	12 [7 - 20]	1.1%	1.0%	9.6 [5.2 - 15.7]	1.0%	0.8%
MPF	17 [11 - 26]	1.5%	1.4%	15 [8 - 22]	1.3%	1.1%	12 [7 - 20]	1.1%	1.0%	9.6 [5.2 - 15.7]	1.0%	0.8%
Fish and seafood	9.7 [4.2 - 16.9]	2.1%	0.4%	7.9 [3.2 - 15.2]	1.8%	0.3%	7 [3 - 14]	1.7%	0.3%	4.9 [1.8 - 11.0]	1.4%	0.3%
MPF	8.0 [3.5 - 13.5]	1.9%	0.4%	6.1 [2.4 - 11.1]	1.5%	0.3%	5 [2 - 10]	1.4%	0.3%	3.6 [1.3 - 8.2]	1.2%	0.2%
PF	0.0 [0.0 - 0.0]	0.0%	0.0%	0.0 [0.0 - 0.0]	0.0%	0.0%	0.0 [0.0 - 0.1]	0.0%	0.0%	0.0 [0.0 - 0.1]	0.0%	0.0%
UPF	0.0 [0.0 - 1.9]	0.2%	0.1%	0.0 [0.0 - 3.0]	0.3	0.1%	0.0 [0.0 - 2.7]	0.3%	0.1%	0.0 [0.0 - 2.0]	0.2%	0.0%
Dairy products	384 [218 - 579]	20.6%	6.4%	410 [240 - 602]	22.8%	6.6%	401 [242 - 587]	23.6%	6.5%	380 [226 - 564]	25.2%	6.6%
MPF	290 [138 - 468]	11.6%	3.6%	307 [155 - 488]	12.4%	3.6%	301 [154 - 482]	12.6%	3.5%	282 [140 - 453]	12.7%	3.3%
PCI	1.7 [0.8 - 3.3]	0.2%	0.1%	1.8 [0.8 - 3.5]	0.2%	0.1%	1.8 [0.9 - 3.4]	0.2%	0.1%	1.7 [0.8 - 3.1]	0.2%	0.0%
PF	25 [13 - 40]	6.2%	1.9%	30 [18 - 47]	7.5%	2.2%	34 [20 - 50]	8.3%	2.3%	37 [22 - 55]	9.8%	2.6%
UPF	49 [25 - 83]	2.6%	0.9%	49 [25 - 80]	2.6%	0.8%	47 [23 - 76]	2.5%	0.7%	44 [22 - 70]	2.5%	0.7%
Miscellaneous[b]	88 [53 - 139]	7.5%	4.6%	75 [44 - 124]	6.7%	3.9%	64 [38 - 106]	6.0%	3.3%	52 [31 - 88]	5.3%	2.8%
MPF	60 [30 - 112]	5.8%	3.8%	51 [25 - 100]	5.4%	3.3%	42 [21 - 85]	4.8%	2.8%	33 [16 - 68]	4.1%	2.3%
PCI	17 [8 - 34]	1.7%	0.8%	14 [6 - 28]	1.4%	0.6%	13 [6 - 25]	1.3%	0.5%	12 [6 - 24]	1.3%	0.5%
**Other foods**												
Alcoholic beverages	53 [6 - 184]	3.1%	3.6%	57 [9 - 176]	3.2%	3.6%	63 [10 - 18]	3.4%	3.8%	78 [14 - 203]	3.9%	4.1%
PF	28 [1 - 134]	2.3%	2.8%	30 [2 - 123]	2.3%	2.8%	33 [3 - 130]	2.5%	2.9%	42 [5 - 151]	2.8%	3.2%
UPF	0.9 [0.0 - 11.5]	0.8%	0.8%	1.3 [0.0 - 12.5]	0.9%	0.8%	1.5 [0.0 - 13.7]	1.0%	0.9%	1.8 [0.0 - 15.7]	1.0%	0.9%
Drinking water	292 [133 - 561]	0.5%	0.5%	309 [140 - 589]	0.5%	0.5%	299 [131 - 562]	0.4%	0.4%	263 [113 - 505]	0.3%	0.4%
MPF	292 [133 - 561]	0.5%	0.5%	309 [140 - 589]	0.5%	0.5%	299 [131 - 562]	0.4%	0.4%	263 [113 - 505]	0.3%	0.4%

BWC blue water consumption, *GHGE* greenhouse gas emissions, *hPDI* healthful plant-based diet index, *MPF* unprocessed/minimally processed foods, *PCI* processed culinary ingredients, *PF* processed foods, *Q* quartile, *UPF* ultra-processed foods.

[a]Variables are adjusted for total energy intake, expressed per 2000 kcal, and presented as median with interquartile range [25 - 75th percentile].

[b]Mixed dishes that contain substantial amounts of animal sourced ingredients (e.g. soups, pizza, and mayonnaise). Supplemental Table 1 displays detailed information on included food items.

Supplemental Table 5 Associations between the PDI indices and all-cause mortality risk by UPF consumption in the EPIC-NL cohort.

			hPDI											Measure of additive interaction	
			Q1 (≤ 50 points)		Q2 (50-54 points)		Q3 (54-58 points)		Q4 (> 58 points)		Per 10-point increase		**P value for trend**[a]		
			HR	95% CI	HR	95% CI	HR	95% CI	HR	95% CI	HR	95% CI		RERI[b,d]	95% CI
**UPF**	Q4 (> 433 g/2000 kcal)	Model 1[c]	1.00	Reference	0.86	0.73, 1.02	0.90	0.74, 1.09	0.82	0.66, 1.04	0.91	0.80, 1.02	0.053	-0.015	-0.075, 0.045
		Model 2[d]	1.00		0.88	0.74, 1.04	0.93	0.76, 1.13	0.86	0.68, 1.08	0.93	0.82, 1.05	0.135		
	Q3 (328-433 g/2000 kcal)	Model 1[c]	0.89	0.77, 1.04	0.78	0.66, 0.91	0.69	0.57, 0.82	0.68	0.56, 0.81	0.82	0.73, 0.92	0.003		
		Model 2[d]	0.91	0.79, 1.06	0.83	0.71, 0.98	0.73	0.61, 0.88	0.76	0.63, 0.92	0.88	0.78, 0.99	0.035		
	Q2 (251-328 g/2000 kcal)	Model 1[c]	0.92	0.79, 1.08	0.73	0.62, 0.86	0.71	0.60, 0.83	0.68	0.58, 0.80	0.81	0.73, 0.91	<0.001		
		Model 2[d]	0.91	0.78, 1.07	0.77	0.65, 0.91	0.77	0.65, 0.90	0.78	0.66, 0.92	0.87	0.78, 0.98	0.054		
	Q1 (≤ 251 g/2000 kcal)	Model 1[c]	1.01	0.85, 1.19	0.83	0.71, 0.97	0.82	0.70, 0.95	0.70	0.61, 0.81	0.83	0.75, 0.93	<0.001		
		Model 2[d]	0.98	0.83, 1.16	0.84	0.71, 0.98	0.85	0.73, 1.00	0.78	0.66, 0.89	0.91	0.81, 1.02	0.024		
			**uPDI**											**Measure of additive interaction**	
			Q1 (≤ 49 points)		Q2 (49-54 points)		Q3 (54-58 points)		Q4 (> 58 points)		Per 10-point increase		**P value for trend**[a]		
			HR	95% CI	HR	95% CI	HR	95% CI	HR	95% CI	HR	95% CI		RERI[b,d]	95% CI
**UPF**	Q4 (> 433 g/2000 kcal)	Model 1[c]	1.00	Reference	1.02	0.81, 1.28	1.12	0.89, 1.41	1.20	0.97, 1.48	1.10	0.97, 1.23	0.078	0.007	-0.018, 0.031
		Model 2[d]	1.00		1.04	0.83, 1.30	1.15	0.92, 1.44	1.17	0.95, 1.46	1.08	0.96, 1.22	0.105		
	Q3 (328-433 g/2000 kcal)	Model 1[c]	0.90	0.72, 1.13	0.95	0.76, 1.17	0.92	0.73, 1.16	0.89	0.70, 1.13	1.02	0.90, 1.14	0.898		
		Model 2[d]	0.97	0.78, 1.22	1.03	0.83, 1.28	0.96	0.76, 1.20	0.91	0.72, 1.15	0.97	0.86, 1.09	0.430		
	Q2 (251-328 g/2000 kcal)	Model 1[c]	0.88	0.71, 1.09	0.83	0.67, 1.03	0.99	0.79, 1.24	0.94	0.73, 1.20	1.13	1.01, 1.27	0.080		
		Model 2[d]	0.97	0.78, 1.19	0.90	0.72, 1.11	1.02	0.81, 1.28	0.96	0.75, 1.23	1.10	0.98, 1.23	0.192		
	Q1 (≤ 251 g/2000 kcal)	Model 1[c]	0.88	0.72, 1.08	0.97	0.79, 1.20	0.96	0.76, 1.21	1.18	0.92, 1.50	1.17	1.05, 1.30	0.006		
		Model 2[d]	0.95	0.77, 1.16	1.01	0.82, 1.25	0.96	0.76, 1.22	1.13	0.88, 1.45	1.08	0.98, 1.20	0.186		

CI confidence interval, *HR* hazard ratio, *hPDI* healthful plant-based diet index, *Q* quartile, *RERI* relative excess risk due to interaction, *uPDI* unhealthful plant-based diet index, *UPF* ultra-processed foods.

[a]Linear trend test was conducted by assigning median values to each quartile and entering this as continuous variables in the models.

[b]Relative excess all-cause mortality risk due to interaction between a 10-point decrease in the hPDI score or 10-point increase in the uPDI score and a 100 g/2000 kcal increase in UPF consumption. The hPDI was recoded into a risk factor for correct calculation of the RERI.

[c]Adjusted for age, sex, and total energy intake.

[d]Adjusted for age, sex, total energy intake, educational level, smoking status, physical activity level, and alcohol consumption.
